# CapG promotes resistance to paclitaxel in breast cancer through transactivation of PIK3R1/P50

**DOI:** 10.7150/thno.36338

**Published:** 2019-09-21

**Authors:** Yayun Chi, Jingyan Xue, Sheng Huang, Bingqiu Xiu, Yonghui Su, Wei Wang, Rong Guo, Lei Wang, Lun Li, Zhiming Shao, Wei Jin, Zhaohui Wu, Jiong Wu

**Affiliations:** 1 Department of Breast Surgery, Breast Cancer Institute, Shanghai Cancer Center, Department of Oncology, Shanghai Medical College, Fudan University, Shanghai, China.; 2 Department of Pathology, Fudan University, Shanghai Cancer Center, Shanghai, China.; 3 Center for Cancer Research, University of Tennessee Health Science Center, Memphis, TN, USA.; 4 Department of Pathology and Laboratory Medicine, University of Tennessee Health Science Center, Memphis, TN, USA.; 5 Collaborative Innovation Center for Cancer Medicine, Department of Oncology, Shanghai Medical College, Fudan University, Shanghai, China.

**Keywords:** breast cancer, chemotherapy resistance, CapG, PIK3R1, CBP/P300

## Abstract

**Background:** Chemotherapy resistance is a major problem in breast cancer treatment and a leading cause of mortality in breast cancer patients. Biomarkers for chemotherapy resistance is under investigation.

Methods: Paclitaxel resistant cells were established and subjected to RNA sequencing. Analysis combined with two additional RNA-seq datasets was conducted. CapG expression in patients with adjuvant chemotherapy was studied in breast cancer resection specimens using IHC and related to pathological response and disease-free survival. Paclitaxel resistance was assessed by half-maximal inhibitory concentrations (IC50) and a mouse xenograft model.

**Results:** Increased expression of actin-binding protein CapG strongly correlated with the resistance to paclitaxel chemotherapy and decreased probability to achieve pathological complete response in breast cancer patients. Overexpressing CapG significantly enhanced paclitaxel resistance in breast cancer cells and xenograft tumors. High CapG level also significantly correlated with shorter relapse-free survival as well as hyper-activation of PI3K/Akt signaling in breast cancer patients. Mechanistically, CapG enhanced PIK3R1 expression which led to increased PI3K/Akt activation. Unexpectedly, CapG was found to bind to the variant-specific promoter of PIK3R1/P50 and directly enhance its transcription. We also identified p300/CBP as a transcriptional coregulator of CapG, which is recruited to PIK3R1 promoter through interaction with CapG, thereby increasing PIK3R1/P50 transcription by enhancing histone H3K27 acetylation. Consistently, inhibiting p300/CBP substantially decreased CapG-dependent upregulation of PIK3R1/P50 and subsequent PI3K/Akt activation, resulting in increased sensitivity to paclitaxel treatment in breast cancer cells.

**Conclusion:** High CapG levels may predict poor paclitaxel response in breast cancer patients. Targeting CapG-mediated hyperactivation of PI3K/Akt pathway may mitigate resistance to chemotherapy in breast cancer.

## Introduction

Chemotherapy is the standard of care for systemic treatment of breast cancer. Among chemotherapeutic agents, paclitaxel (PTX) has shown great efficacy against breast cancer, especially in triple-negative subtype (TNBC). However, a large number of patients acquire drug resistance eventually. Therapeutic resistance is associated with aggressive clinicopathologic features, such as tumor recurrence and metastases. It significantly diminishes the therapeutic efficacy in breast cancer patients and is a leading cause of cancer-related death. Resistance to paclitaxel has been actively explored and a variety of mechanisms have been reported, including upregulation of ATP transporters, mutations or activation of DNA repair system and dysregulation of oncogenic signaling pathways (e.g. Notch, PI3K/AKT, and NF-κB signaling) 1. However, there is an urgent and unmet clinical need for reliably predicting tumor responses to taxane chemotherapy before treatment or choosing proper combination treatment when resistance arises.

Macrophage-capping protein (CapG, also known as gCap39 or MCP) is a member of the gelsolin superfamily which plays important roles in regulating actin assembly 2. In contrast to cytoplasmic localization of the other gelsolin family members, CapG resides in both the cytoplasm and the nucleus 3. CapG interacts with the actin filaments and caps the actin barbed ends to ensure the correct filament arrangement and localization, thereby regulating cell differentiation, membrane ruffling, phagocytosis and cell motility 2, 4-6. Moreover, increased CapG expression has been found in several metastatic cancers, suggesting its role in cancer cell invasion and metastasis 7-9. Intriguingly, the nuclear CapG plays a much stronger role in eliciting invasion than the cytoplasmic counterpart 10, which was speculated to correlate with a potential role of CapG in regulating gene expression 11-13. The *CAPG* gene shares sequence homology with genes encoding basic helix-loop-helix-family DNA-binding proteins, such as *MYC*, suggesting that it may directly bind to chromatin and modulate transcription^14^. Nevertheless, whether the nuclear CapG directly regulates gene transcription and its pathological roles in promoting breast cancer progression are largely unknown.

In this study, we show for the first time that CapG contributes to chemotherapy resistance especially paclitaxel resistance in breast cancer by epigenetically enhancing *PIK3R1/P50* transcription, resulting in increased activation of PI3K pathway. CapG overexpression leads to increased proliferation and paclitaxel resistance in breast cancer cells. In breast cancer patients, high CapG levels are significantly associated with poor response and short relapse-free survival (RFS) after chemotherapy treatment. Mechanistically, CapG binds to chromatin and enhances transcription of *PIK3R1*. The upregulation of PIK3R1, especially its p50α isoform, substantially augments the activation of PI3K/Akt pathway, resulting in increased cell proliferation and decreased apoptosis in breast cancer cells upon paclitaxel treatment. Moreover, CapG interacts with CBP/P300 at the promoter region of PIK3R1, which enhances local histone H3K27 acetylation, leading to increased transcription of *PIK3R1/P50*. Importantly, the correlation between CapG level and PI3K/Akt activation is confirmed in breast cancer patient samples and xenograft animal models. Altogether, our findings support that CapG may serve as a novel predictor of taxane chemotherapy outcome in breast cancer patients, as well as a potential therapeutic target for mitigating resistance to chemotherapies.

## Materials and Methods

### Patients and tumor samples

Informed consent forms were signed by each participant and appropriate ethical committee approval was obtained. For the tissue microarray, 200 primary breast cancer tissue samples from female invasive ductal carcinoma patients (no co-morbidities reported) who received chemotherapy were randomly collected at the Department of Breast Surgery of the Fudan University Shanghai Cancer Center (FDUSCC, Shanghai, P.R. China) between 2002 and 2006. The median follow-up time was 96 months (84-141 months). Prior to constructing the tissue microarray (TMA), each paraffin-embedded tumor sample was defined and the tumor regions marked based on H&E staining. The TMA sections were generated by the Department of Pathology at the FDUSCC. The TMA included duplicate cores from different areas of the same tumor to compare the staining patterns.

### Cell lines, plasmids and transfection

MCF-7, MDA-MB-231, ZR75-1 and T47D cell lines were obtained from ATCC which were characterized by Short Tandem Repeat (STR) profiling. Cells resuscitated from original passage and passaged within 6 months were used in all experiments. All these cells were cultured under standard conditions. Paclitaxel resistant MDA-MB-231 cells (MDA231-PTX) were established by long-term culture in the presence of PTX. MDA231 PTX-10 indicates the sub-clone survived in the culture medium with 10 uM PTX. MDA231 PTX-20 indicates the sub-clone survived in the culture medium with 20 uM PTX. PCDH-CapG, pCMV-myc-p85α and pCMV-myc-p50α were cloned from the MCF-7 cDNA. eGFP-NES-CapG was generated by fusing FragminP-NES to CapG N-terminus as reported 15. All plasmids were transfected with Lipofectamine 2000 (Invitrogen).

### Chromatin Immunoprecipitation (ChIP)

ChIP assays were carried out as described previously 16. Briefly, treated cells were cross-linked with 1% formaldehyde, sheared to an average size of 500 base pairs, and then immunoprecipitated with antibodies as indicated. The ChIP-qPCR primers were designed according to the ChIP-Seq data to amplify the putative CapG binding region within PIK3R1. For ReChIP experiments ReChIP buffer (Dilution Buffer, 10mM DTT) was added to beads following the first IP and incubated at 37°C for 50 minutes. The sample was then diluted 40 times and subject to 2^nd^ immunoprecipitations.

### Electrophoretic Mobility Shift Assay (EMSA)

The EMSA assay was carried out as previously published 17. Briefly, GST-CapG was purified from bacterial lysates using glutathione-agarose beads (Amersham Biosciences) and its concentration was quantified using the Pierce bicinchoninic acid (BCA) protein assay kit (Thermo Fisher Scientific). The single-stranded complementary oligonucleotide fragments (80bp/fragment with 20bp overlap) corresponding to PIK3R1/P50 promoter regions (-742- -482bp) were synthesized (Genewiz) and biotinylated using a biotin 3-end DNA labeling kit (Thermo Fisher Scientific). The biotinylated complementary oligonucleotide pairs were annealed to make double-stranded biotin-labeled probes and then phenol chloroform purified. EMSAs were performed according to the instructions provided for the LightShift Chemiluminescent EMSA kit (Thermo Fisher Scientific).

### CCK-8 assay

The cell proliferation Ability was assessed by CCK-8 assay. CCK8 analysis was subsequently performed as per the standard procedures. Briefly, the breast cancer cells were seeded in 96-well plates and grown for 8 h. The cells were then treated with various concentrations of PBS solution containing PTX, with each concentration tested in triplicate, and then cells were cultured for an additional 3-6 days. The IC50 values were obtained by using the Logit method and reported as the mean ± SD from three independent determinations.

### Xenografts study

All animal studies were conducted in accordance with a protocol approved be the Institutional Animal Care and Use Committee at FUSCC. MCF-7/CapG or MCF-7/PCDH cells (1x10^7^) were injected into the mammary fat pads of BALB/c nude mice. 14 days after tumor cell transplantation, the mice were injected intraperitoneally with paclitaxel (20 mg/kg; Sigma) every 4 days for six cycles. Tumor sizes were measured with caliper and calculated by the formula V = *(W)^2^*x* L/2*. At end point, the mice were sacrificed and the mammary tumors were harvested for further analyses.

### Bioinformatics analyses

Gene expression data of breast cancer cases from The Cancer Genome Atlas (TCGA) breast dataset were downloaded from TCGA website (https://www.cancer.gov/tcga). The patients were divided into two groups according to the median CAPG mRNA expression (high versus low group). For RNA‐Seq analysis of breast cancer cell lines (CAPG knock-down versus control), the raw sequencing reads were aligned to the human genome (release version hg19) using TopHat. The raw read counts were filtered and normalized using the voom method through the R package limma. The R package limma was also used for gene differential expression (DE) analysis. P values were corrected using FDR (false discovery rate) with an alpha value set to 0.05. The differentially expressed genes (log2FC>1 or log2FC<-1, and p<0.05) from TCGA database and our own RNA-seq data were used for further analysis. The Gene Ontology (GO) enrichment and Kyoto Encyclopedia of Genes and Genomes (KEGG) pathway analysis were further applied to enrich the microarray data using DAVID 6.8 databases (https://david.ncifcrf.gov/summary.jsp) and KOBAS 3.0 databases (http://kobas.cbi.pku.edu.cn/anno_iden.php).

### Statistical analyses

Correlations between clinical-pathological parameters and interested markers were evaluated using contingency tables and Pearson χ2 test or Fisher's exact test. Disease-free survival and overall survival were derived from the Kaplan-Meier estimate and compared by the log-rank test. Statistics was analyzed using SPSS (version 13.0; SPSS Company). The other results were presented as means ± S.D. and analyzed with Student's t test. All P values are two-sided and a P value less than 0.05 was considered statistically significant.

## Results

### CapG promotes paclitaxel resistance in breast cancer cells

**Figure 1:**
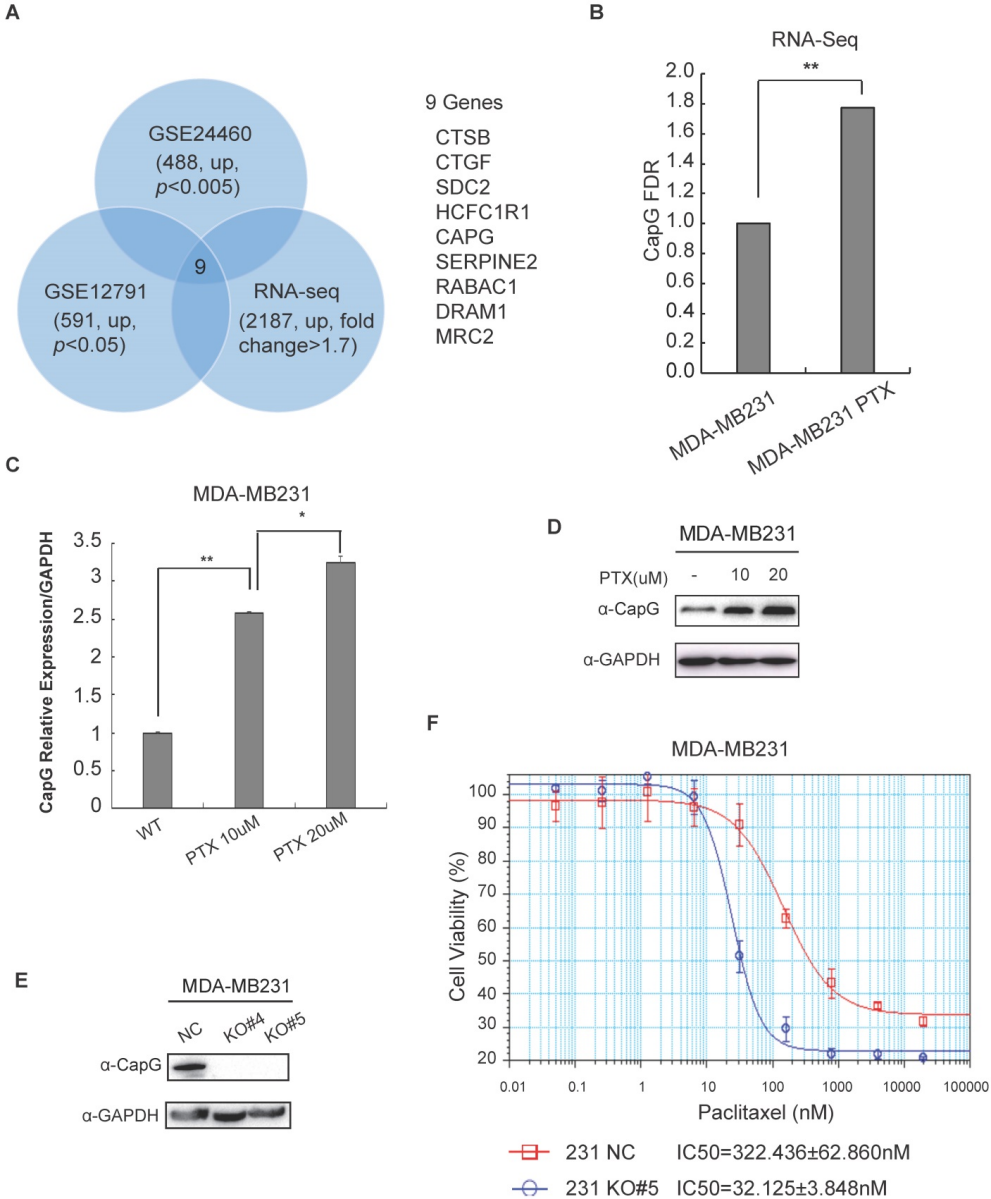
CapG renders breast cancer cells resistant to paclitaxel. (**A**) PTX resistance-associated candidate genes were selected through analyzing three breast cancer datasets as indicated. Nine overlapped genes were identified as shown. (**B**) CapG transcription was measured by qPCR in PTX-resistant MDA-MB231 cells and its parental cells. **P<0.01. (**C**) The mRNA level of CapG was measured by qPCR in MDA-MB231 cells resistant to indicated dosages of PTX. *P<0.05, **P<0.01. (**D**) Total cell extracts from different PTX-resistant MDA-MB231 sub-clone cells were immunoblotted as shown. (**E**) Two clones of CapG knockout MDA-MB231 cells generated with CRISPR/Cas9 and parent MDA-MB231 cells were analyzed by immunoblotting. (**F**) Cell proliferation was determined by SRB assay after treatment with different concentrations of paclitaxel for 48 h. Mean concentrations of paclitaxel that induce the 50% of cell death (IC50) on wild-type (NC) and CAPG knockout (KO#5) MDA-MB-231 cells were calculated and compared.

In order to examine the genes involved in resistance to taxane chemotherapy, paclitaxel resistant MDA-MB-231 cells (MDA-PTX) were established and subjected to RNA sequencing. Analysis combined with two additional RNA-seq datasets from chemotherapy-resistant breast cancer cells (GSE24460 18 and GSE12791 19) revealed that there were 9 common genes among the three datasets whose expression was significantly increased compared with their respective wild type cells (Figure 1A). Interestingly, several genes, such as DRAM1, SERPINE2, SDC2 and CTSB, have been shown to correlate with chemotherapy efficiency in cancers 20-24. Moreover, CTGF signaling pathway are well-known as an important modulator of breast cancer response to Taxol, and CTGF increases drug resistance to paclitaxel by upregulating survivin expression in human osteosarcoma cells 25-27. The upregulation of these genes were further validated in MDA-MB-231 PTX resistant cells by quantitative PCR. We found that 7 out of 9 genes, including CAPG, were substantially increased in PTX-resistant MDA-MB-231 cells compared with wild type cells (Figure 1C, S1A). As we recently showed that CapG enhances breast cancer metastasis 28, we decided to focus our further investigation on the role of CAPG in promoting PTX resistance.

CAPG gene transcription was significantly increased in breast cancer cells acquired resistance to chemotherapy (Figure 1B, C; S1B, C). In accordance, the protein level of CapG was also increased in PTX-resistance cells (Figure 1D). To validate the correlation between CapG and chemosensitivity, we depleted CapG in MDA-MB231 cells with CRISPR/Cas9 (Figure 1E) and examined their sensitivity to PTX treatment. We found that the half-maximal inhibitory concentrations (IC50) of paclitaxel in CAPG-KO MDA-MB231 cells (32.125±3.848 nM) was approximately 1/10 of that of the parental MDA-MB231 cells (322.436±62.860 nM) with the p value 0.0012 (Figure 1F). Consistently, CapG depletion in paclitaxel resistant cells significantly enhanced their sensitivity to PTX (Figure S1D, E). Moreover, overexpression of CapG also significantly increased the sensitivity to PTX in MCF-7 cells (Figures S1F). All these data suggest that increased CAPG levels render breast cancer cells more resistant to PTX treatment. This notion was further corroborated by our findings that CapG knockout enhanced the apoptosis of MDA-MB231 cells upon PTX treatment (Figure S2A), which is accompanied by decreased cells proliferation and reduced cell survival in response to PTX treatment (Figure S2B, 2C). Consistent results were also observed in PTX-resistant MDA-MB231 cells with CapG depletion (Figure S2D, 2E). Altogether, these results indicate that CapG promotes resistance to PTX in breast cancer cells and knocking down of CapG was able to mitigate PTX resistance in breast cancer.

### CapG correlates with pathological complete response (pCR) and disease-free survival (DFS) in breast cancer patients

**Figure 2:**
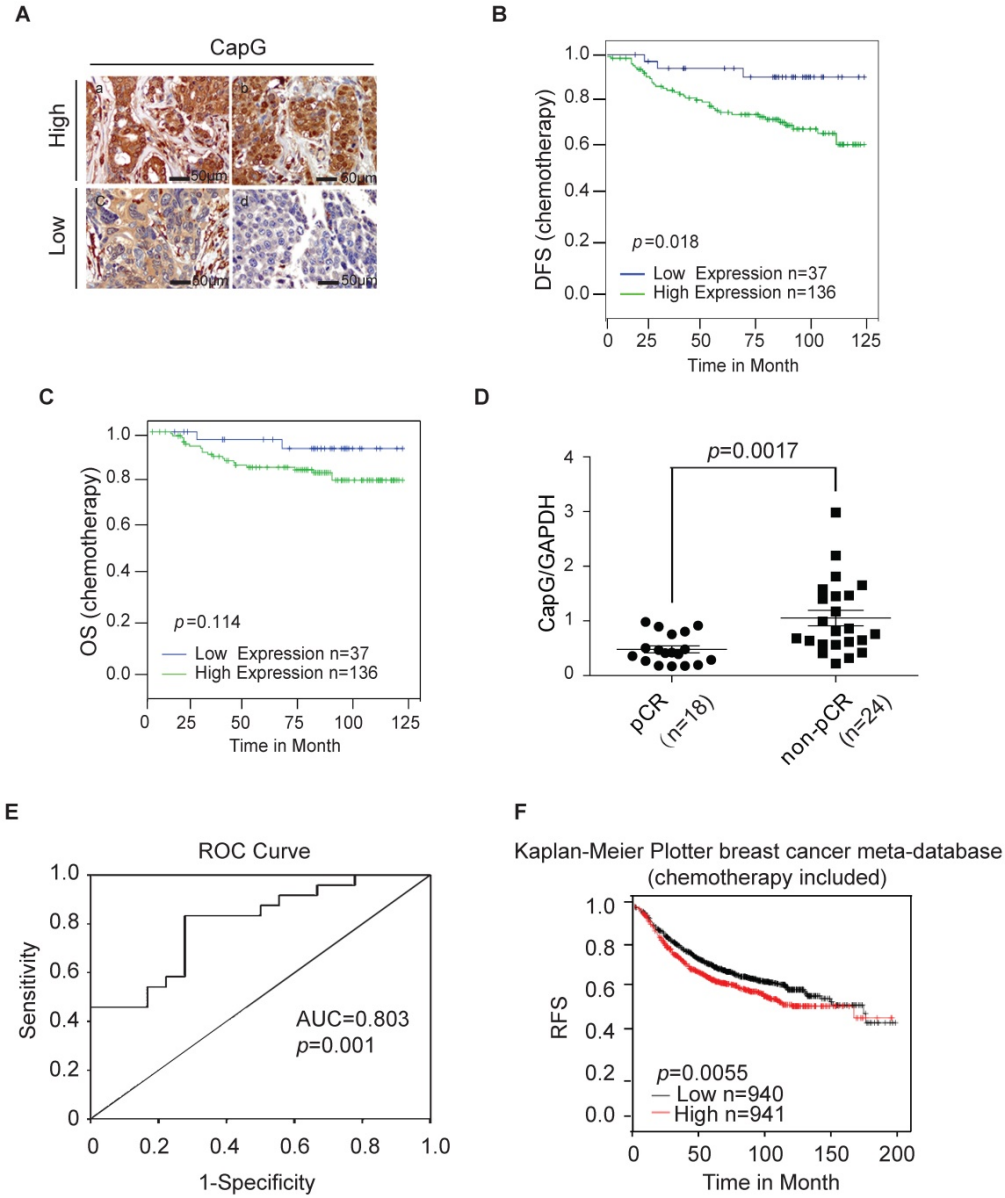
CapG correlates with pathological complete response (pCR) and disease-free survival (DFS). (**A**) CapG expression was determined by IHC staining in the tissue array from breast cancer patients who received PTX-based chemotherapy. (**B**) Correlation between CapG expression and disease-free survival (DFS) was analyzed with Kaplan-Meier curves in this cohort. (**C**) Correlation between CapG expression and overall survival (OS) was analyzed with Kaplan-Meier curves in the same cohort. (**D**) Expression levels of CapG in breast cancer patients who received PTX-based neoadjuvant chemotherapy (n=42, 18 pCR vs 24 non-pCR), were quantitated by real-time PCR. GAPDH was used as an internal control. (**E**) Receiver operating characteristic (ROC) curve of 42 breast cancer patients' level of CAPG was used for analyzing the area under the curve (AUC) value. (**f**) High expression of CapG associated with poor RFS following chemotherapy treatments in 1881 breast cancer patients retrieved from the Kaplan-Meier Plotter breast cancer meta-database (kmplot.com, 2014 version).

To further evaluate the clinical significance of CapG in breast cancer chemotherapy resistance, we assessed the level of CapG with immunohistochemistry (IHC) assay in a tissue array with a cohort of 200 primary breast cancer patients treated with chemotherapy (Figure 2A). The relationship between CAPG and clinical-pathological characteristics was assessed as shown in Table S1. CAPG was negatively correlated with estrogen receptor (ER) (p=0.026) and positively correlated with human epidermal growth factor receptor 2 (HER2) (p=0.050) in this patient cohort, while it displayed no relationship with other status. Moreover, high CapG significantly associated with poor disease-free survival (DFS, p=0.018) (Figure 2B). High CapG was also related with decreased overall survival (OS), although the p value was not statistically significant (p=0.114) (Figure 2C). Univariate analysis demonstrated that high CAPG expression in cancer tissue (HR=3.746; 95% CI: 1.157-12.134; p=0.028), along with tumor stage (HR=1.982; 95% CI: 1.194-3.292; p=0.008) and axillary lymph node stage 3 (HR=3.782; 95% CI: 1.521-9.403; p=0.004) were significant unfavorable prognostic factors for DFS in BC patients (Table S2). Multivariate analysis also demonstrated that high CAPG expression (HR=3.348; 95% CI: 1.029-10.894; p=0.045) and tumor stage (HR=1.888; 95% CI: 1.151-3.094; p=0.005) were independent unfavorable prognostic factors for DFS in BC patients and only tumor stage (HR=2.728; 95% CI: 1.475-5.043; p=0.001) and axillary lymph node stage (HR=2.087; 95% CI: 1.417-3.047; p<0.001) were independent unfavorable prognostic factors for OS in BC patients (Table S2).

To further validate the association between CapG and poor response to PTX, an independent set of 42 patients who received neoadjuvant chemotherapy with PTX regimen (18 pCR patients and 24 non-pCR patients) was further analyzed by quantitative RT-PCR. The clinicopathological features of this validation set are listed in Table S2. Consistently, we found the mRNA level of CAPG was significantly higher in tumors from pCR patients than that in non-pCR patients (P = 0.0017) (Figure 2D), confirming that the lower CAPG expression is associated with a better response to PTX regimen chemotherapy. To evaluate the predictive value of CapG in PTX response in breast cancer patients, we used the receiver operating characteristics (ROC) curve to analyze the sensitivity and specificity of CAPG. The area under the curve (AUC) was 0.803 (confidence interval = 0.672-0.935; P=0.001), which indicated high accuracy of predictive value. The sensitivity and specificity were 83.3 and 72.2%, respectively (Figure 2E). Furthermore, we queried the Kaplan-Meier Plotter breast cancer database and found that, in breast cancer patients received systemic chemotherapy, patients with high CapG expression exhibited significantly decreased RFS interval than those with low CapG expression (P=0.0055, HR=1.25; 95% CI, 1.07-1.46) (Figure 1F). These findings strongly suggest that the increased CapG expression significantly associated with decreased sensitivity to PTX chemotherapy, and may predict decreased chance of achieving pCR in breast cancer patients receiving PTX regimen chemotherapy.

### High CapG level is correlated with the activation of PI3K/Akt signaling pathway in breast cancer

**Figure 3:**
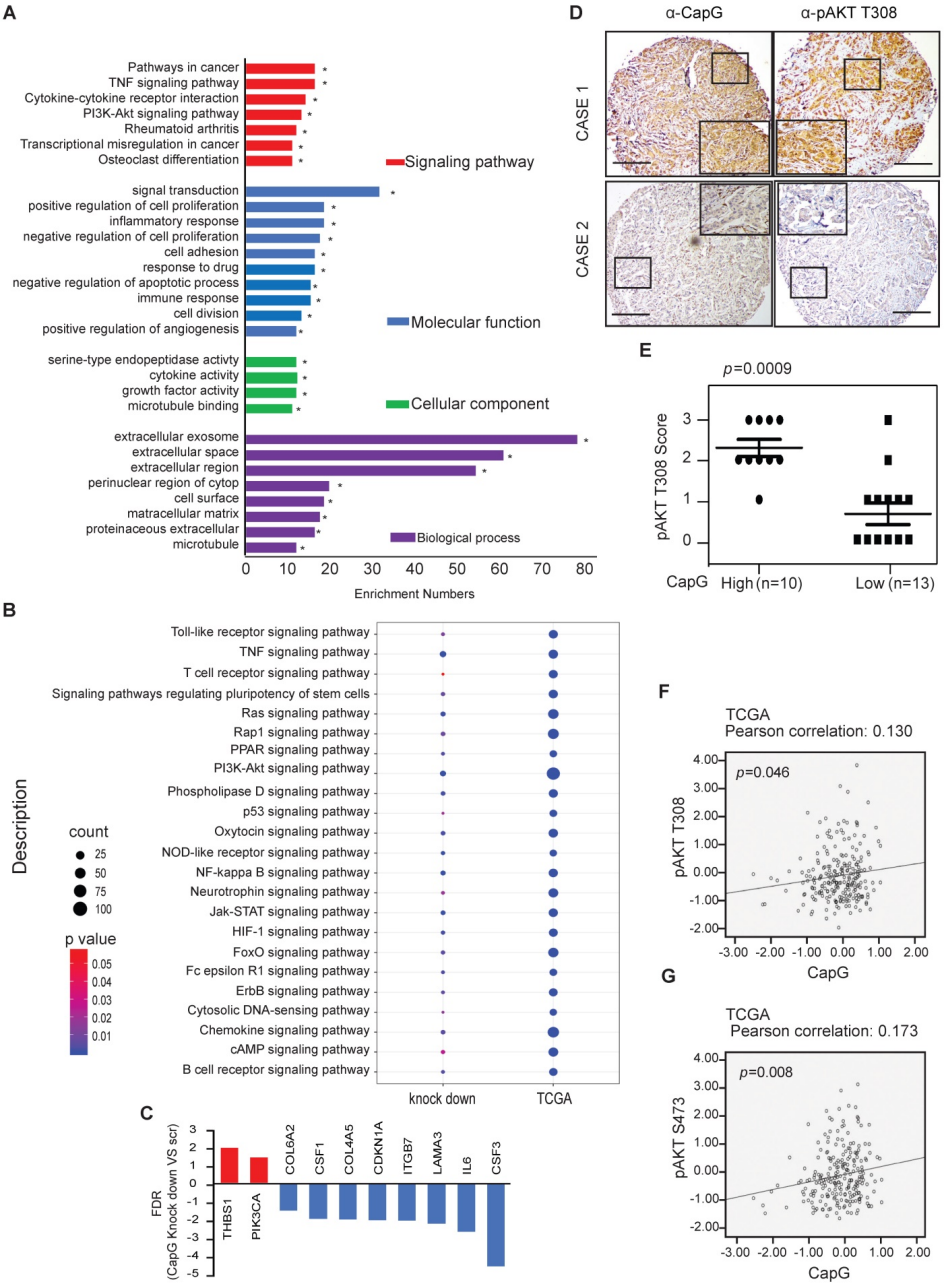
High CapG is correlated with the activation of PI3K/Akt signaling pathway in breast cancer. (**A**) Functional annotation clustering of genes altered by CapG knocked down in MDA-MB231 cells is shown. Significantly enriched groups nominated by the gene ontology term are ranked based on the group enrichment scores. Red indicates signaling pathway; blue, molecular functional; green, cellular component; purple, biological process.** (B)** The relationship between CapG and indicated signaling pathway-related genes in CapG knock-down MDA-MB231 cells and TCGA database. Circle surface, the relation index with CapG expression; color, p value. **(C)** CapG knockdown altered transcription levels of a group of genes associated with PI3K-AKT signaling pathway. (**D**) The expression of CapG and Akt pT308 were visualized by IHC staining in breast cancer samples. Scale bar: 400 μm. (**E**) Plots of Akt pT308 levels in 23 samples of breast cancer stratified by the CapG level. (**F-G**) Pearson analysis of gene expression data and protein phosphorylation data from breast cancer patients (n=348, TCGA-RPPA) was used for depicting the correlation between CapG and AKT pT308 (**F**) or AKT pS473 (**G**).

To better understand how CapG promotes drug resistance in breast cancer cells, RNA-seq was performed to analyze the gene expression profile affected by CapG knockdown. Gene set enrichment analysis showed that CapG knockdown affected multiple signaling pathways, such as the TNF signaling pathway, the chemokine signaling pathway, the PI3K/AKT signaling pathway and response to drug, resulting in remarkable changes in the biological processes and cellular components of the growth factor and cytokine activity (Figure 3A). Further analysis showed that CapG mRNA level was positively correlated with PI3K/AKT signaling pathway. Consistent observation was also obtained in TCGA database 29, 30 (Fig.3B). Moreover, we found the mRNA levels of PI3K/AKT signaling-related genes were indeed regulated by CapG in MDA-MB231 cells (Figure 3C). Considering that PI3K/Akt signaling activation has been shown to promote therapy resistance in breast cancers 31-33, these data suggested that CapG may promote drug resistance through modulating PI3K/Akt signaling pathways in breast cancer cells.

To further validate whether CapG expression affected activation of PI3K/Akt pathway, CapG protein level was assessed in breast cancer tissue array. We observed a significant increase of Akt phosphorylation in the CapG high expression group compared with the CapG low group (Figure 3D and 3E), suggesting that CapG upregulation may enhance PI3K/Akt signaling activation. Consistently, CapG mRNA level positively correlated with Akt phosphorylation at both Thr308 (p=0.046) and Ser473 (p=0.008) residues in the breast cancer samples which contains 68 triple negative samples, 42 HER2 positive samples, 151 luminal A samples, 83 luminal B samples and 4 healthy controls from the TCGA-RPPA database (Figure 3F and 3G). In parallel, overexpression of CapG also significantly increased the transcription of PI3K pathway downstream target genes *CCND1* and *MYC* in ZR75-1 cells (Figure S3A). In support of this observation, CapG expression level was also positively correlated with expression of *CCND1* and *MYC* in breast cancer patients (GSE2990) 34. (Figure S3B and S3C Taken together, these data strongly support that CapG promotes PI3K/Akt signaling activation, which may play a role in CapG-dependent drug resistance in breast cancers.

### Nuclear CapG activates PI3K/Akt signaling pathway

**Figure 4:**
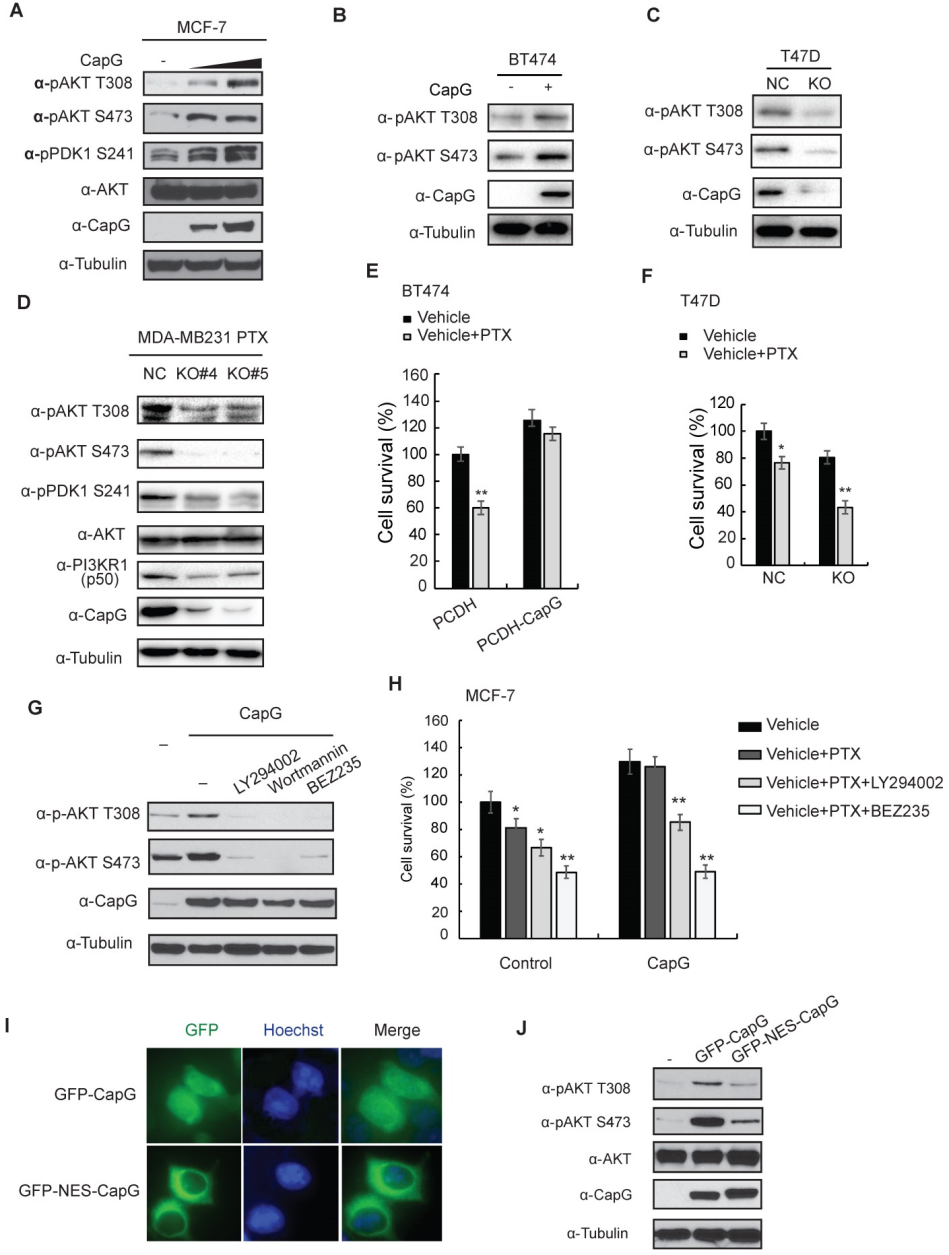
Nuclear CapG activates PI3K/Akt signaling pathway. (**A**) MCF-7 cells transfected with increasing CapG construct were immunoblotted as shown. (**B**) BT474 cells transfected with increasing CapG construct were immunoblotted as shown.** (C)** CapG was deleted with CRISPR/cas9 system in T47D cells and immunoblotted as shown. Parent cells (NC) were used as control**. (D)** CapG was deleted with CRISPR/cas9 system in PTX-resistant MDA-MB231 cells and two stable clones were immunoblotted as shown. Parent cells (NC) were used as control.** (E)** Cell survivals was examined by CCK-8 assay in BT474 cells transfected with CapG construct.** (F)** Cell survivals was detected by CCK-8 assay in T47D CapG-KO cells. (**G**) MCF-7 cells were transfected with CapG as shown. Cells were mock treated or treated with PI3K inhibitors LY294002 (25 uM), Wortmannin (100 nM) or BEZ235 (100 nM). Whole cell lysates were immunoblotted as shown. (**H**) MCF-7 or MCF-7/CapG stable cells were treated with paclitaxel alone or along with LY294002 or BEZ235 for 48 h. The cell survival was examined and data from three independent experiments were pooled and shown as mean ± S.D. *P <0.05, **P <0.01. (**I**) MCF-7 cells were transfected with EGFP-CapG or EGFP-NES-CapG. Subcellular localization of CapG was visualized by GFP. (**J**) MCF-7 cells were transfected with EGFP-CapG or EGFP-NES-CapG and total cell extracts were analyzed by immunoblotting as indicated.

To further explore the mechanisms involved in CapG-promoted PI3K/Akt signaling, we ectopically expressed CapG in breast cancer cells and examined its impact on PI3K/Akt activation. Overexpression of CapG substantially increased the phosphorylation of Akt (T308 and S473) and its upstream kinase PDK1 (S241) in a dose-dependent manner in MCF-7 cells (Figure 4A). The similar results was obtained in BT474 cells (Figure 4B). In contrast, PI3K signaling was inhibited in MDA-MB231 cells by depleting CapG with shRNA in MDA-MB231 cells as well as in CapG-KO PTX-resistant MDA-MB-231 cells and T47D CapG-KO cells (Figure 4C-D and S4A). PI3K signaling recovered with ectopic overexpression of CapG in MDA-MB231 CapG-KO cells (Figure S4B). The sensitivity to paclitaxel was changed along with the PI3K signaling in these cells (Figure 4E-F and S4B). In addition, the increased Akt phosphorylation by CapG was abrogated by PI3K inhibitors (Figure 4G), which also attenuated CapG-enhanced MCF-7 and T47D cell survival upon paclitaxel treatment (Figure 4H and S4C). All this body of evidence strongly suggests that CapG may enhance PI3K/Akt activation, thereby promoting paclitaxel resistance in breast cancer cells.

Intriguingly, it was reported that the nuclear localized CapG played a critical role in eliciting cell invasion, and a transcription regulatory role of CapG was implied 10. Consistent with a previous report 15, CapG localizes in both nuclear and cytoplasmic subcellular compartment (Figure 2A, top row). Moreover, N-terminal fusion of a nuclear export sequence (NES) to CapG was sufficient to drive its nuclear export and diminish nuclear CapG fraction in MCF-7 cells (Figure 4I). Surprisingly, Akt activation by NES-CapG was significantly decreased compared to that by wild type CapG (Figure 4J), suggesting nuclear localization of CapG is critical for activating PI3K/Akt signaling in breast cancer cells.

### CapG binds to the promoter of PIK3R1/P50 and promoted its transcription

Nuclear CAPG has been suggested to modulate gene transcription, potentially by controlling actin nucleation and assembly, which is involved in regulating gene transcription 11-13. We also reported previously that CapG enhanced the transcription of the pro-metastatic gene STC-1, contributing to increased metastasis in BC 28. The RNA-seq data showed that PIK3CA was changed in the MDA-MB231 CapG knock-out cells (Figure 3C). So we first validated whether PIK3CA expression was regulated by CapG. The qPCR analysis and immunoblotting data showed almost no influence of PIK3CA expression by CapG in MCF-7 CapG stable cells or MDA-MB231 CapG-KO cells (Figure S5). To examine whether nuclear CapG is involved in transcription regulation and its potential impact on PI3K activation, we carried out a chromatin immunoprecipitation followed by next generation sequencing (ChIP-Seq) analysis of CapG and mapped its chromatin-binding regions in human genome. Interestingly, ChIP-Seq results revealed a CapG binding region (chr5:67,583,461-67,583,707) within *PIK3R1* gene body but not *PIK3CA* gene body (Figure S6A). We aligned this binding region with PIK3R1 genomic sequence in UCSC genome browser and found that it located near the transcription start site of the PIK3R1 variant 3 (P50). Moreover, this CapG-binding region co-localizes with a CpG island and a Pol II-binding region identified in MCF-7 cells by ENCODE, suggesting that this CapG/Pol II-binding region may serve as a promoter for regulating PIK3R1/P50 transcription. This notion was further supported by the histone H3K27 acetylation distribution surrounding this region (Figure S6A). All these findings suggest that CapG may regulate PIK3R1/P50 transcription by binding to this genomic region.

**Figure 5:**
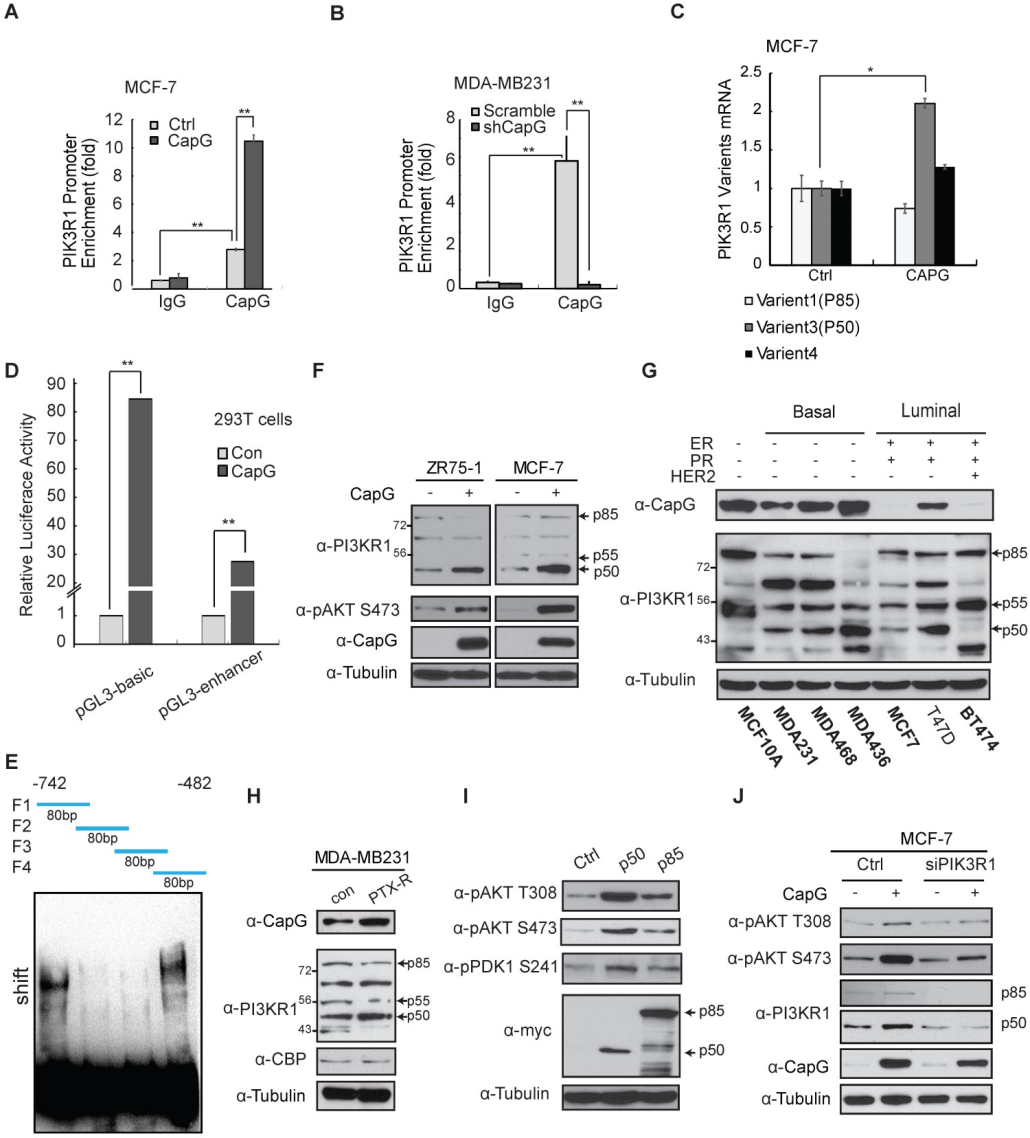
CapG binds to the promoter of PIK3R1/P50 and enhances its transcription. (**A**) ChIP analyses of CapG recruitment to PIK3R1/P50 promoter were performed in MCF-7 cells transfected with control or CapG. **P<0.001. (**B**) Similar ChIP analyses as in (**A**) were done in MDA-MB-231 or MDA-MB-231 CapG-depleted cells. (**C**) Expression of PIK3R1 variants was analyzed by qPCR in MCF-7 cells transfected with control or CapG. *P< 0.05. (**D**) 293T cells were transfected with control or CapG plasmid along with PI3KR1/P50 promoter- luciferase reporter (-742bp—482bp) as shown. The activity of Renilla/Firefly luciferases was assayed by the dual-luciferase reporter assay system and normalized luciferase activity was showed as mean ±s.d. **P <0.01. (**E**) Four 80bp fragments (F1, F2, F3, F4) were synthetized, with a 20bp overlap between each pair of fragments (as shown on the top). Specific oligonucleotides interaction with purified CapG protein was determined using gel shift assay. (**F**) MCF-7 cells and ZR75-1 cells were transfected with CapG and cell lysates were analyzed by immunoblotting as shown. (**G**) Whole lysates of breast cancer cell lines and MCF10A cells were immunoblotted as shown. (**H**) Matched sub-clones of wild type MDA-MB231 and the respective PTX-resistant (PTX-R) cells were lysed and immunoblotted as indicated. (**I**) MCF-7 cells were transfected with control, p50α or p85α plasmids, and analyzed by immunoblotting as shown. (**J**) MCF-7 cells were transfected with CapG alone or along with siPIK3R1. Total cell lysates were immunoblotted as indicated.

We validated the chromatin-binding of CapG on PIK3R1/P50 promoter in MCF-7 and MDA-MB-231 cells, which was enhanced or decreased by overexpressing CapG in MCF-7 cells or CapG depletion in MDA-MB-231 cells, respectively (Figure 5A and 5B). In T47D cells, CapG binding on PIK3R1/P50 promoter region was also increased along with increased CapG expression (Figure S6B). Moreover, the transcription of PIK3R1 variant 3 (P50), but not other variants, was significantly increased upon CapG overexpression in MCF-7 cells (Figure 5C). Consistently, knocking down CapG also predominantly down-regulated the transcription of PIK3R1/P50 (Figure S6C). In addition, the induction of PIK3R1/P50 was significantly reduced in cells transfected with NES-CapG compared to wild type CapG, supporting a critical role of nuclear CapG in promoting PIK3R1/P50 transcription (Figure S6d). Moreover, overexpression of CapG significantly upregulated the expression of luciferase reporters (pGL3-basic and pGL3-enhancer) fused to the promoter region of P50 (Figure 5d), suggesting CapG may bind to the P50 promoter region to drive it transcription. To further map the exact region revealed by ChIP-Seq data for this bind, GST-CapG protein was purified and EMSA assay was carried out. Four 80bp fragments within this region (chr5:67,583,461-67,583,707) were synthetized and biotin-labeled, with a 20bp overlap between each pair of fragments. The biotin-labeled probes including F1 fragment and F4 fragment could form a complex with GST-CapG but not the other two fragments. These results further corroborated that CapG could directly bind to the F1 (-742 to -692) region and F4 (-532 to -482) region within P50 promoter to regulate its expression (Figure 5E).

In line with the transcription analyses, ectopic CapG remarkably increased the protein levels of PIK3R1/p50α, whereas the other variants were minimally affected in MCF-7 and ZR75-1 cells (Figure 5F). Then CapG expression level was detected in a series of breast cancer cells (Figure 5G and S6E). In accordance, we found the expression level of CapG correlated with PIK3R1/p50α levels in a panel of breast cancer cells (Figure 5G). Similar correlation was also observed in tumors from breast cancer patients (Figure S6F). Furthermore, we found in paclitaxel-resistant MDA-MB-231 cells, PIK3R1/p50α expression was augmented along with increased CapG level, compared with that in the wild type counterpart (Figure 5H). These results indicate that CapG-mediated PIK3R1/ p50α up-regulation may play a critical role in promoting paclitaxel resistance in breast cancer cells.

To determine whether PIK3R1/p50α is sufficient for enhancing PI3K/Akt activation, we overexpressed PIK3R1/p50α or full length PIK3R1/p85α in MCF-7 cells. Transfection of either p85α or p50α increased PDK1 and Akt activation (Figure 5I). Intriguingly, ectopic p50α induced more robust activation of PDK1 and Akt than that by p85α, which may be attributed to the lack of the inhibitory N-terminal SH3-BH domain in the p50α variant 35, 36. Meanwhile, activation of PI3K pathway by CapG was abrogated by PIK3R1 knockdown (Figure 5J), supporting that PIK3R1 induction, especially the p50α variant, is required for CapG-promoted PI3K signaling activation.

### CapG promotes PIK3R1/P50 transcription by recruiting CBP/p300 to enhance H3K27 acetylation

**Figure 6:**
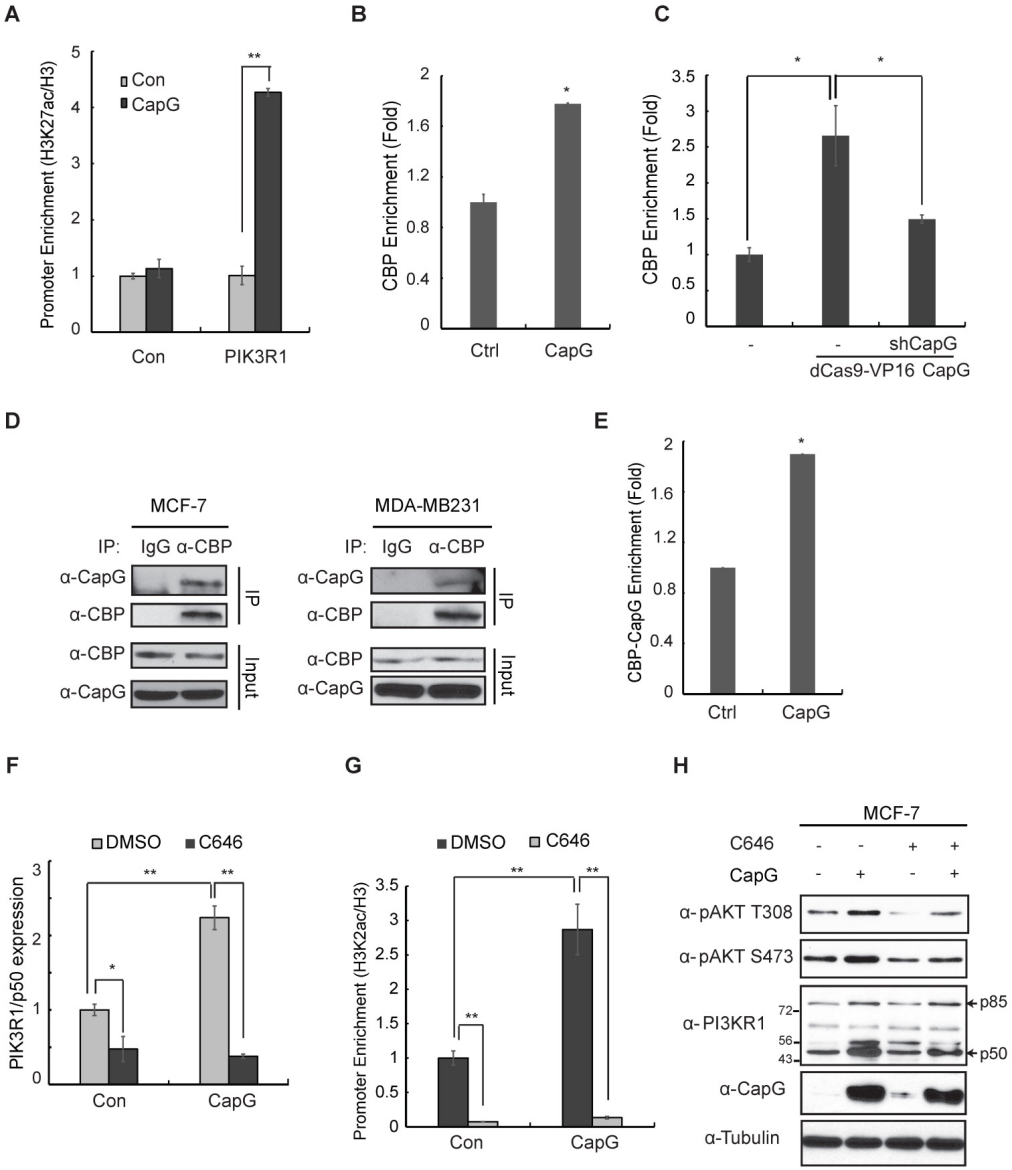
CapG facilitates CBP/p300 binding at PIK3R1/P50 promoter and enhances H3K27 acetylation. (**A**) ChIP analysis of histone H3K27 acetylation around the CapG-binding site within PIK3R1 genomic sequence was carried out in MCF-7 cells transfected with control or CapG. A neighboring region (chr5: 68,288,104-68,288,123) was used as control. Histone H3 ChIP was used for normalization, and data were shown as percentage of H3K27ac enrichment. (**B**) CBP recruitment to the PIK3R1/P50 promoter was analyzed by ChIP in MCF-7 cells transfected with CapG. (**C**) Similar CBP ChIP analysis as in (**B**) was carried out in dCas9-VP16-CapG activated MCF-7 cells transfected with control or shCapG. (**D**) MCF-7/CapG and MDA-MB231 cell lysates were immunoprecipitated with the anti-CBP or control. The precipitates were immunoblotted as indicated. (**E**) ChIP-reChIP analyses by sequential IP with anti-CapG and anti-CBP were carried out in MCF-7 cells transfected with CapG. (**F**) Expression of PIK3R1/P50 was analyzed by qPCR in MCF-7 cells transfected with control or CapG, in the presence or absence of C646 (20 μM). (**G**) H3K27ac surrounding the CapG-binding region was analyzed by ChIP in MCF-7 cells transfected and treated as in (**F**). (**H**) MCF-7 cells were transfected with CapG and treated with C646 (20 μM) as indicated. Total cell extracts were analyzed by immunoblotting. *P<0.05; ** P<0.01.

We compared the CapG binding region within PIK3R1 genomic sequence with ChIP-Seq data from other transcriptional regulators deposited in the UCSC genome browser. Interestingly, the CapG binding region is marked with enriched histone H3K27 acetylation (H3K27ac) and overlaps with a CBP/EP300 binding region (Figure S6A). These observations prompted us to examine whether CapG could regulate CBP/p300-mediated H3K27 acetylation at the PIK3R1/P50 promoter in breast cancer cells. Indeed, we found H3K27ac at CapG-binding PIK3R1/P50 promoter region was significantly increased by overexpressing CapG (Figure 6A). Moreover, overexpressing CapG in MCF-7 cells also enhanced CBP recruitment on PIK3R1/P50 promoter region (Figure 6B). Consistently, we also detected an increased CBP recruitment on the PIK3R1/P50 promoter in dCAS9/VP16-activating CapG overexpression MCF-7 cells compared with that in MCF-7 cells, which was attenuated by depletion of CapG by shRNA (Figure 6C). We also found p300 binding on the same promoter region of PIK3R1/P50 (Figure S7A). These results suggest that CapG may enhance H3K27ac at PIK3R1/P50 promoter by facilitating CBP/p300 recruitment, thereby promoting PIK3R1/P50 transcription.

By co-immunoprecipitation (co-IP), we confirmed the interaction between CapG and CBP/p300 in MCF-7 and MDA-MB231 cells (Figure 6D, S7B). More importantly, ChIP-reChIP analyses showed that CapG and CBP formed a complex at the promoter region of PIK3R1/P50 (Figure 6E). Furthermore, treatment with a CBP/p300 specific inhibitor C646 was able to abrogate CapG-mediated upregulation of PIK3R1/P50 transcription (Figure 6F), as well as the increased acetylation of H3K27 at the PIK3R1/P50 promoter region (Figure 6G). We also found that attenuated PIK3R1/P50 upregulation by CBP/p300 inhibition correlated with decreased Akt activation by CapG overexpression (Figure 6H). Collectively, these findings strongly indicate that CapG-facilitated enrichment of CBP/p300 at PIK3R1/P50 promoter and increased H3K27 acetylation are essential for induction of PIK3R1/P50 transcription and subsequent PI3K/Akt activation in breast cancer cells.

### CapG enhances breast cancer resistance to paclitaxel treatment *in vivo*

**Figure 7:**
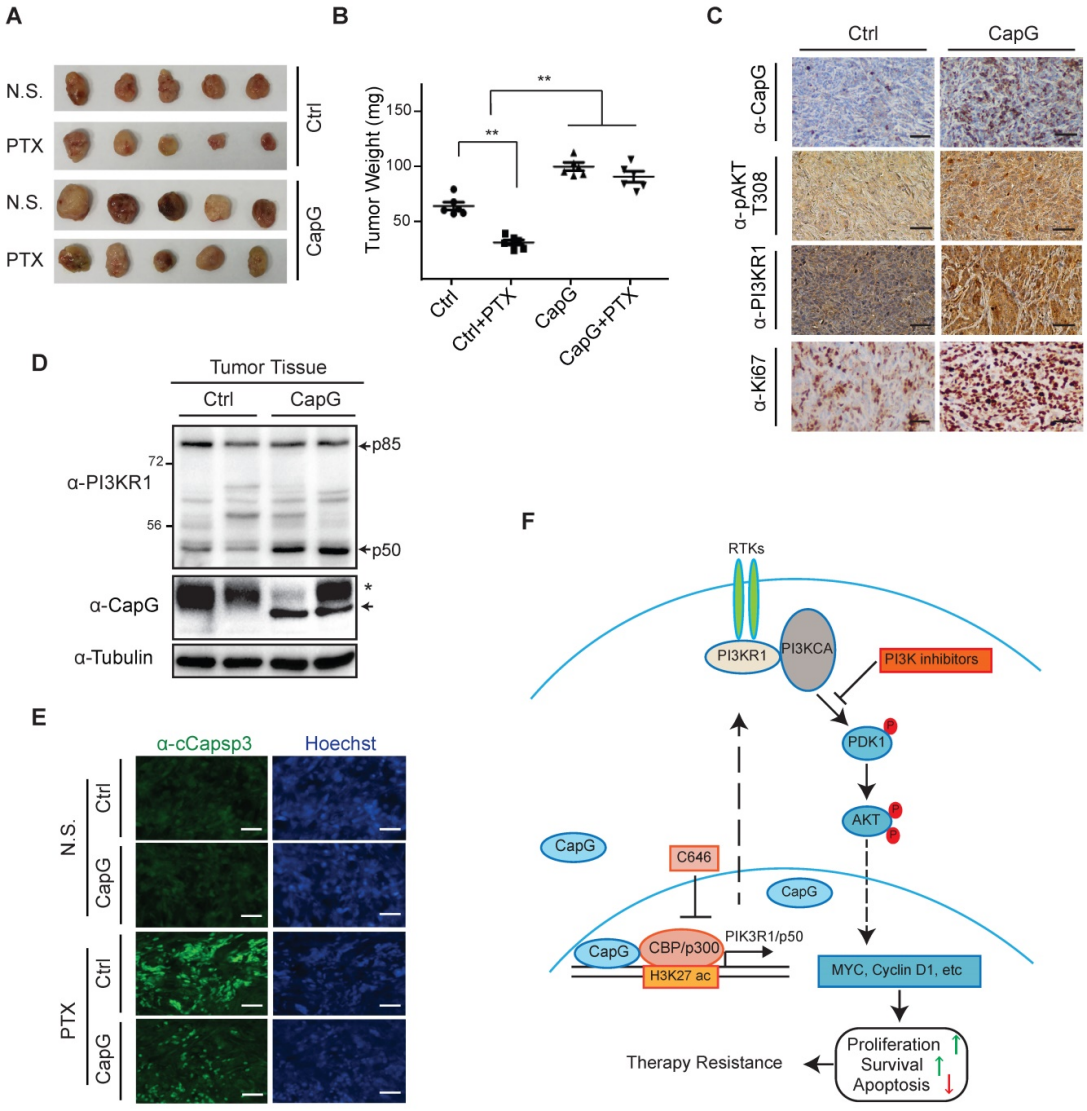
CapG promotes breast cancer resistance to paclitaxel treatment *in vivo*. (**A-B**) Nude mice orthotopically transplanted with MCF-7/vector or MCF-7/CapG cells (n=8) were treated with paclitaxel (20 mg/kg) or N.S. Xenograft tumor growth was monitored and showed as the tumors size (**A**) and harvested tumor weight (**B**). (**C**) Levels of CapG, Akt pT308, PI3KR1 and Ki67 in xenograft tumors were determined by IHC staining. Scale bar: 40 μm. (**D**) Total protein extracts from xenograft tumors were immunoblotted as indicated. *: Non-specific band, arrow: HA-CapG. (**E**) Cleaved Caspase-3 was detected with immunofluorescence staining in tumors. Scale bar: 40 μm. (**F**) A model depicting the potential role of CapG in modulating breast cancer cell response to paclitaxel.

To validate the role of CapG in paclitaxel resistance *in vivo*, we generated orthotopic mammary tumors model with MCF-7/Control or MCF-7/CapG cells. We found CapG overexpression promoted MCF-7 xenograft tumor growth. Moreover, while paclitaxel effectively inhibited the tumor growth in mice implanted with control cells, it barely reduced tumor growth in mice transplanted with MCF-7/CapG cells, indicating a paclitaxel resistance-promoting effect of CapG (Figure 7A-B). Consistently, CapG-overexpressed xenograft tumors exhibited increased PI3KR1 expression, Akt activation (pAkt T308) and proliferation (Ki-67) determined by IHC staining (Figure 7C). Furthermore, the p50 protein level was also confirmed to be increased in MCF-7/CapG xenograft tumors (Figure 7D). The decreased Caspase 3 activation in paclitaxel-treated MCF-7/CapG xenograft tumors, compared with that in MCF-7 xenografts (Figure 7E), further supported an anti-apoptotic role of CapG in paclitaxel-treated breast cancers, likely owing to increased PI3K/Akt activation. All together, these data strongly support that CapG promotes paclitaxel resistance through activating the PI3K/Akt signaling pathway, which enhances proliferation and attenuates apoptosis in breast cancer cells exposed to paclitaxel.

## Discussion

Accumulating evidence indicates that enhanced activation of oncogenic molecular signaling pathways such CDK4/6, HDAC, Src, IGFR-1, FGFR and PI3K/Akt in breast cancer cells plays a significant role in developing therapy resistance. Accordingly, improved response has been observed in patients when combining inhibitors targeting these pathways along with chemotherapy 37, 38. However, the mechanism activating these signaling pathways, such as PI3K/AKT signaling, in cancer cells has yet to be completely understood. Further exploring these underlying mechanisms may reveal novel therapeutic targets for mitigating therapeutic resistance. In this study, we show that CapG promotes resistance to chemotherapy, especially paclitaxel resistance, in breast cancer by activating PI3K signaling. Intriguingly, our results revealed an unexpected role of CapG in directly modulating transcription through epigenetically enhancing H3K27ac at promoter region of PIK3R1P50. This epigenetic regulation was achieved by CapG-facilitated recruitment or stabilization of CBP/P300 at the target gene promoters. We found CapG specifically promoted PIK3R1/P50 isoform transcription in breast cancer cells, which subsequently triggered activation of PI3K/Akt signaling, resulting in increased cell growth and decreased apoptosis in breast cancer cells exposed to paclitaxel (Figure 7F). Importantly, we validated the positive correlation between CapG expression and levels of Akt activation, as well as PI3K/Akt downstream target gene expression in breast cancer patient samples. Moreover, patients with lower CapG level tend to achieve pCR upon paclitaxel treatment in a neoadjuvant setting. Altogether, our findings strongly indicate that high CapG levels may serve as a novel biomarker for predicting primary and acquired resistance to chemotherapy in breast cancer patients. Moreover, PI3K inhibitors or pharmacological inhibition of H3K27ac by CBP/p300 inhibitors may help to counteract CapG-dependent paclitaxel resistance and improve response to chemotherapy.

CapG has been reported to be an oncogene involved in migration and invasion in breast cancer and ovarian cancer 8, 10, 39. Interestingly, NES-fused CapG failed to induce cell invasion 10. Moreover, nuclear CapG was shown to regulate VP16-driven transactivation, suggesting its potential role in regulating gene transcription 10. Additionally, we previously demonstrated that CAPG could bind to STC-1 promoter region (-451 bp to -75 bp) to activate STC-1 transcription and substantially promote breast cancer metastasis 28. Nevertheless, how the nuclear CapG modulates aggressive cell behavior remains unclear. We found NES-fusion substantially decreased CapG-promoted PIK3R1/P50 transactivation and PI3K/Akt activation. Moreover, our ChIP-Seq analysis revealed that CapG could bind to the promoter region of PIK3R1/p50. This finding was further corroborated by the ChIP-reChIP analyses showing that CapG formed a complex with CBP/p300 at promoter region of PIK3R1/p50 gene. Accordingly, the local histone H3K27 acetylation was enhanced by CapG-facilitated CBP/p300 binding, leading to increased PIK3R1/P50 transcription. All these results support a transcription regulatory role of nuclear CapG in promoting activation of PI3K/Akt signaling pathway, thereby contributing to the chemotherapy resistance in breast cancer cells. Nevertheless, whether the actin-capping activity of CapG is involved in therapy resistance in breast cancer cells remains to be further determined. Also, it is plausible that additional genes modulated by CapG may also contribute to the therapy resistance as well as cell invasion, which warrant further investigation.

It has been demonstrated that PI3K pathway is closely linked to chemotherapy resistance in breast cancer 32, 33, 40-42. The elevated phosphorylation of Akt predicts poor outcome among breast cancer patients 43-47. In agreement with these findings, our data showed that CapG significantly increased Akt phosphorylation in breast cancer cells which rendered them resistant to paclitaxel treatment. Consistently, CapG expression positively correlated with the level of Akt phosphorylation in breast cancer TCGA-RPPA dataset. Although somatic mutations of PIK3CA, PIK3R1 and PTEN have been shown to correlate with therapy resistance, the mechanisms of abnormal PI3K pathway activation in therapy-resistant cells have not been fully elucidated. We found that CapG was involved in the transcriptional upregulation of PIK3R1/P50 and subsequent activation of PI3K/Akt pathway. PTEN downregulation was reported to activate PI3K/Akt pathway which may involve prolonged association between PIK3R1/p85α and the IRS-1 as well as with ErbB3, contributing to the therapy resistance in breast cancers 48. Intriguingly, we found CapG predominantly increased the transcription of PIK3R1/P50 variant, which is expected to activate PI3K signaling more efficiently than p85α. Since p50α lacks the N-terminal SH3-BH domain, which inhibits PI3K/p110 catalytic activity, p50α-associated p110 likely will be more active than those associated with p85α 49, 50. Moreover, the N-terminal SH3-BH region of p85α has been shown to bind directly to PTEN and enhance its lipid phosphatase activity, resulting in reduced PI3K activation 35. Therefore, selective upregulating of PIK3R1/p50α, instead of p85α, by CapG may lead to more efficient activation of PI3K/Akt activation in breast cancer cells. Interestingly, PIK3R1/p50α and p55α variants have been shown to be selectively upregulated by STAT3 in mouse mammary epithelial cell line KIM-2, which may inhibit Akt activation during mammary gland involution^51^. Although the mechanisms regulating distinct effects of PIK3R1/p50α in modulating Akt activation in mouse mammary epithelial cells and human breast cancer cells remain to be further delineated, this study supports that different PIK3R1 variants (p85, p55 and p50) could be transcriptionally regulated by variant-specific promoters.

In conclusion, our data demonstrate that high CapG level is associated with poor RFS and non-pCR in breast cancer patients received PTX-based chemotherapy, which at least in part are owing to increased activation of PI3K/Akt pathway. Since hyper-activation of PI3K/Akt signaling has been shown as a shared mechanism responsible for resistance to multiple chemotherapeutic drugs 41, 52-54, we envision that high CapG level may have potential clinical value of predicting poor response to chemotherapy in breast cancer patients. Inhibition of PI3K/Akt pathway by PI3K inhibitors abolished Akt phosphorylation and PTX resistance mediated by CapG. Since inhibiting CBP/p300 by C646 also substantially decreased PI3K/Akt activation by CapG overexpression in breast cancer cells, targeting CBP/p300 may serve as an alternative strategy to counteract CapG-mediated paclitaxel resistance. Taken together, our study supports that CapG may serve as a promising molecular biomarker to predict chemotherapy resistance as well as a potential therapeutic target for improving chemotherapy response in breast cancer.
