# Supplementary material for: CapG promotes resistance to paclitaxel in breast cancer through transactivation of PIK3R1/P50
Source: Theranostics. 2019 Sep 21;9(23):6840–55. doi: 10.7150/thno.36338 (PMC6815964; doi:10.7150/thno.36338)
Supplement: Supplementary file 1 — Supplementary figures and tables. [file thnov09p6840s1.pdf]

Supplementary Data

Supplementary Materials and Methods

Chemicals

Paclitaxel (PTX) was purchased from Selleck. PI-3 kinase inhibitor LY 294002 (9901P) was purchased from CST. BEZ235 (A8246), Wortmannin (A8544) and C646 (42207) were all purchased from APEBio.

Antibodies

Antibodies used were: anti-CapG monoclonal (sc-166428, Santa Cruz), anti-PI3KR1 monoclonal (PA5-29613, ThermoScientific) and anti-pAKT-Thr308 (9542, Cell Signaling Technology), anti-pAKT-Ser473 (4526s, Cell Signaling Technology), anti-AKT (4691, Cell Signaling Technology), anti-Tubulin (CP06, Oncogen), anti-PDK-Ser241 (3438, Cell Signaling Technology), anti-Histone 3 (39134, Active Motif), anti-H3K27ac (GTX128944, GeneTex), anti-CBP (sc-369, Santa Cruz), anti-p300 (54062S, Cell Signaling Technology), light chain Goat anti Mouse (115-035-174, Jackson), light chain Mouse anti Rabbit (211-032-171, Jackson).

Immunoprecipitation and immunoblotting

For immunoprecipitation experiments, cells were lysed in 10% PBS and 90% Lysis buffer (20 mM Tris (pH 7.0), 250mM NaCl, 3 mM EDTA, 3mM EGTA,0.5% NP-40, 2mM DTT, 0.5mM PMSF, 20mM β-glycerol phosphate, 1mM sodium orthovanadate, 1 μg/ml leupeptin, 1 μg/ml aprotinin, 10mM p-nitrophenyl phosphate, 10mM sodium fluoride). In all, 1 μg of indicated antibody or IgG control was added into lysates and incubated with protein G-Sepharose at 4°C overnight.

Sepharose-enriched immunocomplexes were resolved on SDS-PAGE, transferred to polyvinylidene fluoride (PVDF) membrane and analyzed with immunoblotting.

Immunohistochemistry staining

Expression levels of CapG, PI3KR1, pAkt T308 and Ki67 in postoperative paraffin-embedded tumor specimens from breast cancer patients and mice tumor tissues were detected with IHC. The Immunohistochemistry staining was following the protocols described previously. The concentrations of antibodies used are as follows: CapG, 1:200, 1:200, PI3KR1, 1:500 and pAkt T308, 1:200. The Envision and diaminobenzidine (DAB) Color Kit was purchased from Gene Tech Company Limited (Shanghai, China). The staining procedures strictly followed the supplier's recommendation.

Dual luciferase assay

HEKT293T cells were seeded in 96-well plates at 60% confluence. After 24 h, cells were co-transfected with CapG, PIK3R1/P50-Promoter-Luciferase and 2 ng of Renilla luciferase per well using Lipofectamine 3000 (Invitrogen). Total DNA was adjusted to 500 ng with empty pcDNA vector. Compounds were dispensed into each well 6 h after transfection. Then, the cells were cultured for 24 h, and cell lysates were examined for firefly luciferase activity and Renilla luciferase activity with the Dual-Luciferase Reporter Assay Kit (Promega, Beijing, China). Relative transcriptional activity = Firefly Luciferase / Renilla Luciferase.

Oligonucleotides

Primers for ChIP:

PIK3R1 binding Forward 5'-TGCGAGTTGCAATCGACCT-3'

PIK3R1 binding Reverse 5'-AGGGCATCCTCGGACGAATA-3'

PIK3R1 control Forward 5'-CTTCCTGTGTTTGGCTGTGC-3'

PIK3R1 control Reverse 5'-CAAGTGGCCAATGCAAGACC-3'

Primers for qPCR:

PIK3R1 isoform 1 Forward 5'-GCCACGGTACAATCAGACGA-3'

PIK3R1 isoform 1 Reverse 5'-GAATCCAGGCTGACTCTCACA-3'

PIK3R1 isoform 3 Forward 5'-TGGCCCACTTGGTGGAAGAA-3'

PIK3R1 isoform 3 Reverse 5'-TTGGTGGTTTAGGAGGCAGTG-3'

PIK3R1 isoform 4 Forward 5'-CTCTGCTACTGCGCCGACA-3'

PIK3R1 isoform 4 Reverse 5'-TCCCCCTTCCCAAAGCTAACA-3'

PIK3CA primer 1 Forward 5'- TAGGCAAGTCGAGGCAATGG-3'

PIK3CA primer 1 Reverse 5'- CTGGTCGCCTCATTTGCTCA-3'

PIK3CA primer 2 Forward 5'- CGACTTTGTGACCTTCGGCT-3'

PIK3CA primer 2 Reverse 5'- GGCATGCCGATAGCAAAACC-3'

CCND1 Forward 5'-TTCGGGATGATTGGAATAGC-3'

CCND1 Reverse 5'-TGTGAGCTGGCTTTGAG-3'

MYC Forward 5′-CTTCTCTCC GTCCTCGGATTCT-3′

MYC Reverse 5′-GAAGGTGATCC AGACTCTGACCTT-3′

GAPDH Forward 5'-AATGTCACCGTTGTCCAGTTG-3'

GAPDH Reverse 5'-GTGGCTGGGGCTCTACTTC-3'

Oligonucleotide fragments for EMSA:

PIK3R1-F1: attgccactttctcaaataaagggcaaacatccgcttagaaaggtatgcccttctctgcccttctatcctttaccccatt

PIK3R1-F2: cttctatcctttaccccattagctggcacataaacctaatgcagagacaccttttaattagcaggtcatatggccattta

PIK3R1-F3: gcaggtcatatggccatttaaatgcgagttgcaatcgacctcataaaaatagccgcagtgttgttttattctgaatcgtc

PIK3R1-F4: ttgttttattctgaatcgtcacatgatcatggagtatagtatgcatatggtgtggggtttaatattcgtccgaggatgcc

Supplementary Figure Legends

Figure S1. CapG renders breast cancer cells resistant to paclitaxel**.** (**A**) qPCR analysis of mRNA expression in two dose of MDA-MB-231 PTX sub-clone cells. **P<0.01. (**B**) CapG expression was analyzed in GSE24460 and GSE12791 database **(C)**. **(D)** CapG was knocked out by CRISPER/Cas9 system in MDA-MB PTX cells and its expression was determined by immunoblotting. (**E**) IC50 was determined by survival fraction measured with Cell Proliferation Reagent WST-1 (Roche) in MDA-MB231 PTX cells and MCF-7 stable cells (**F**).

Figure S2. Depletion CapG expression reduces MDA-MB231 proliferation and survival in response to PTX treatment**.** (**A**) MDA-MB-231stable cells were treated with or without PTX for 48 h and apoptosis was analyzed with FACS assay. Data from three independent experiments were pooled and shown as mean±s.d. **P<0.01, ***P<0.001. (**B**) The same number of MDA-MB231 wild cells and CapG knock out cells were seeded in 96 well plate and incubated in the live cell imaging system for 4 days. The cell proliferation rate from three independent experiments were

pooled and shown as mean ±s.d. *P<0.05. **(C)** The same number of MDA-MB231 wild cells and CapG knock out cells were seeded in 96 well plate and cultured with or without PTX in the culture medium. After incubated in the live cell imaging system for 4 days, the cell survival rate from three independent experiments were calculated and shown as mean ±s.d. *P<0.05, **P<0.01. The same experiment was carried out in MDA-MB231 PTX cells and CapG knock out cells and shown in **(D)** and **(E)**.

Figure S3. CapG level is correlated with PI3K/Akt signaling pathway related genes in breast cancer. **(A)** qPCR analyses of CCND1 and MYC expression in breast cancer cells transfected with control or CapG. **P< 0.01. **(B-C)** Pearson analysis of gene expression data from breast cancer patients (GSE2990) was used for depicting the correlation between CapG and CCND1 (B) or MYC (C).

Figure S4. CapG activates PI3K/Akt signaling pathway and leads to paclitaxel resistance. **(A)** MDA-MB231 cells were transfected with increasing shCapG plasmid. Cell lysates were immunoblotted as shown. **(B)** CapG was recovered by transfection with CapG constructs in MDA-MB231 CapG-KO cells and immunoblotted as shown. Cell survivals was detected by CCK-8 assay and showed below. **(C)** T47D cells were transfected with empty vector or CapG plasmids. 12 hrs later, they were treated with paclitaxel alone or along with LY294002 or BEZ235 for 48 h. The cell survival was examined and data from three independent experiments were pooled and shown as mean ± S.D. *P <0.05., **P <0.01.

Figure S5. PIK3CA expression is not regulated by CapG. **(A)** Expression of PIK3CA was analyzed with two pairs of specific primers by qPCR in MCF-7 cells transfected with control or CapG. **(B)** Expression of PIK3CA was analyzed with two pairs of specific primers by qPCR in

MDA-MB231 KO cells. **(C)** MCF-7 cells transfected with control or CapG and MDA-MB231 KO cells were harvest and immunoblotted as shown.

Figure S6. CapG binds to the promoter of PIK3R1/p50 and promotes its transcription. (**A**) Representative illustration of ChIP-Seq-identified CapG binding region within PIK3R1 gene and corresponding transcription regulatory elements retrieved from UCSC genome browser. H3K27ac ChIP-Seq data were retrieved from GM12878 cells. Pol II (POLR2A) ChIP-Seq data were from MCF-7 cells. (**B**) ChIP analyses of CapG recruitment to the PIK3R1/P50 promoter region was carried out in T47D and T47D-CapG cells. **P<0.01. (**C**) qPCR analysis of PIK3R1 variants mRNA expression in MDA-MB-231 scramble cells and shCapG stable cells. *P< 0.05. (**D**) qPCR analysis of PIK3R1/P50 mRNA expression in MCF-7 cells transfected with control, GFP-CapG or GFP-NES-CapG plasmids. *P< 0.05. **P<0.01. **(E)** HER2 positive breast cancer cell lines were analyzed by immunoblotting as shown. (**F**) Breast cancer tissues were analyzed by immunoblotting as shown.

Figure S7. CapG interacts with CBP/p300. (**A**) ChIP analyses of p300 binding to PIK3R1/P50 promoter were performed in MDA-MB231 cells. **P<0.01. (**B**) MDA-MB231 cell lysates were immunoprecipitated with the anti-p300 or control. The precipitates were immunoblotted as indicated.

Supplementary Table Legends

Table S1. Relationship between CapG expression and clinicopathological features in 200 primary breast cancer patients with chemotherapy for IHC detection.

Table S2. Univariate and multivariate Cox regression analyses of DFS and OS in BC patients.

Table S3. The clinicopathological information of 42 patients who received neoadjuvant chemotherapy with PTX regimen (18 pCR patients and 24 non-pCR patients).

sFigure 1

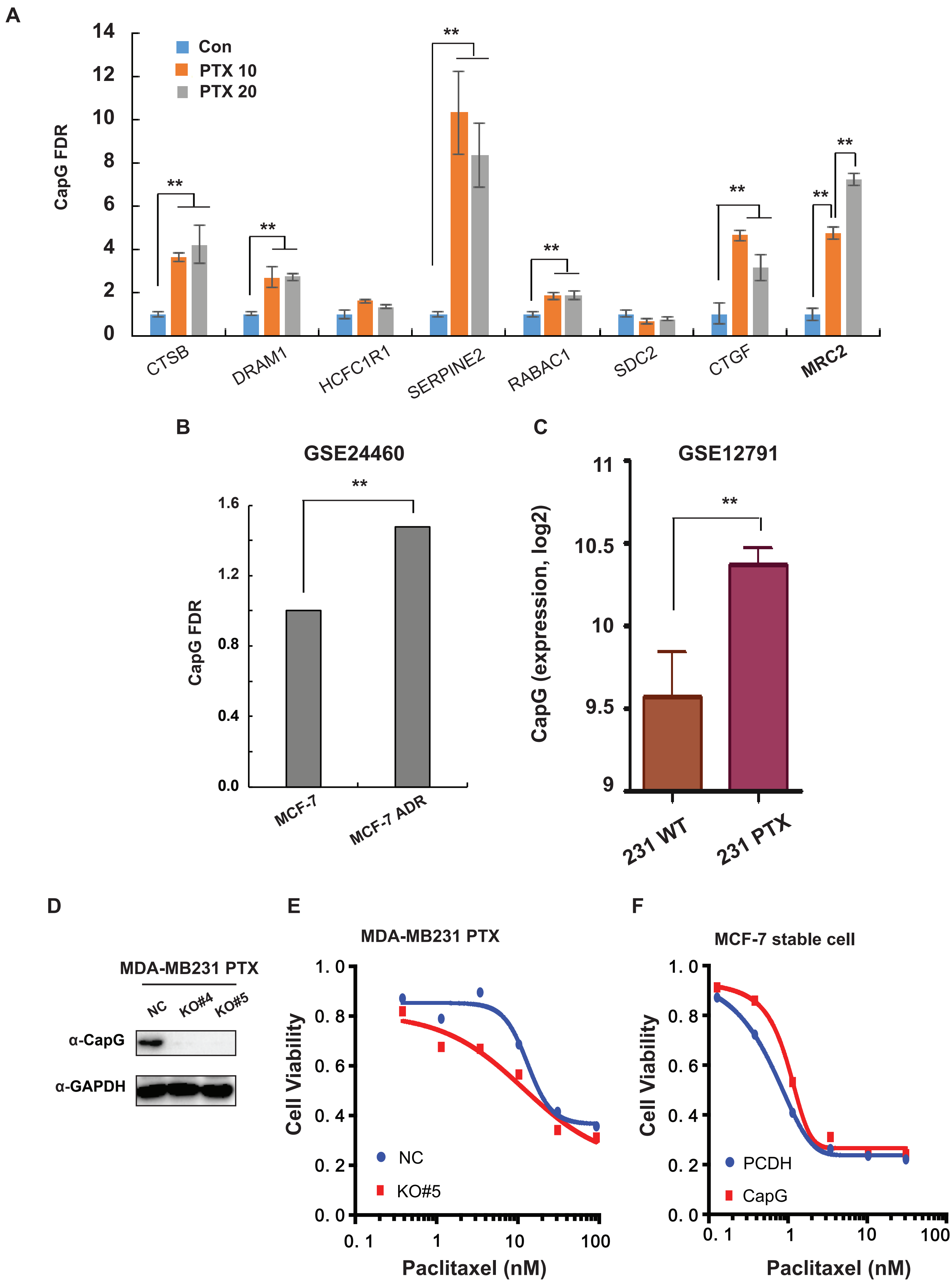
A
Con
PTX 10
PTX 20
CapG FDR
14
12
10
8
6
4
2
0
**
CTSB
DRAM1
HCFC1R1
SERPINE2
RABAC1
SDC2
CTGF
MRC2
B
GSE24460
CapG FDR
1.6
1.2
0.8
0.4
0.0
MCF-7
MCF-7 ADR
C
GSE12791
CapG (expression, log2)
11
10.5
10
9.5
9
231 WT
231 PTX
D
MDA-MB231 PTX
NC
KO#4
KO#5
α-CapG
α-GAPDH
E
MDA-MB231 PTX
Cell Viability
NC
KO#5
Paclitaxel (nM)
F
MCF-7 stable cell
Cell Viability
PCDH
CapG
Paclitaxel (nM)

sFigure 2

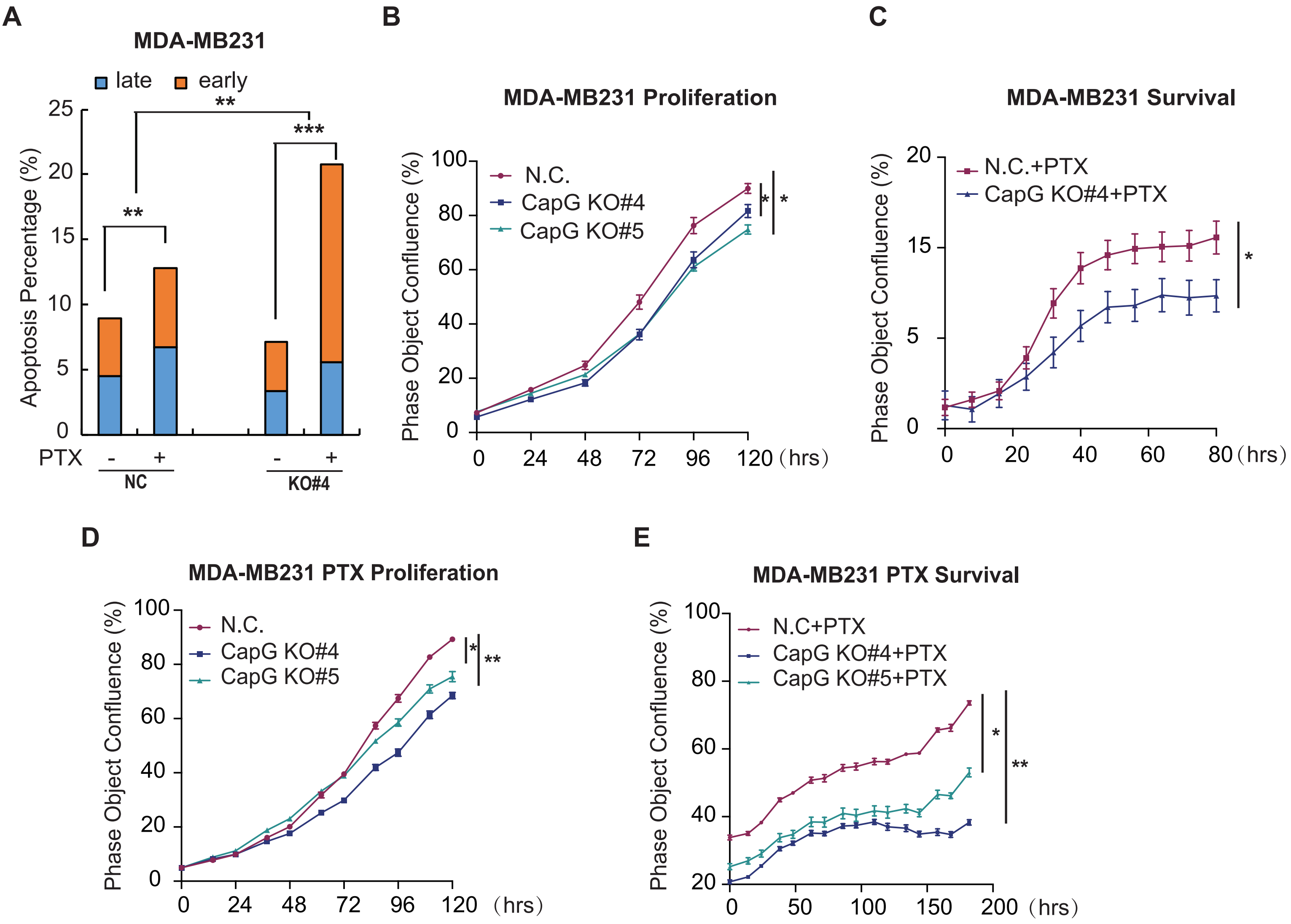
A
MDA-MB231
late
early
Apoptosis Percentage (%)
PTX
NC
KO#4
B
MDA-MB231 Proliferation
N.C.
CapG KO#4
CapG KO#5
Phase Object Confluence (%)
(hrs)
C
MDA-MB231 Survival
N.C.+PTX
CapG KO#4+PTX
Phase Object Confluence (%)
(hrs)
D
MDA-MB231 PTX Proliferation
N.C.
CapG KO#4
CapG KO#5
Phase Object Confluence (%)
(hrs)
E
MDA-MB231 PTX Survival
N.C+PTX
CapG KO#4+PTX
CapG KO#5+PTX
Phase Object Confluence (%)
(hrs)

sFigure 3

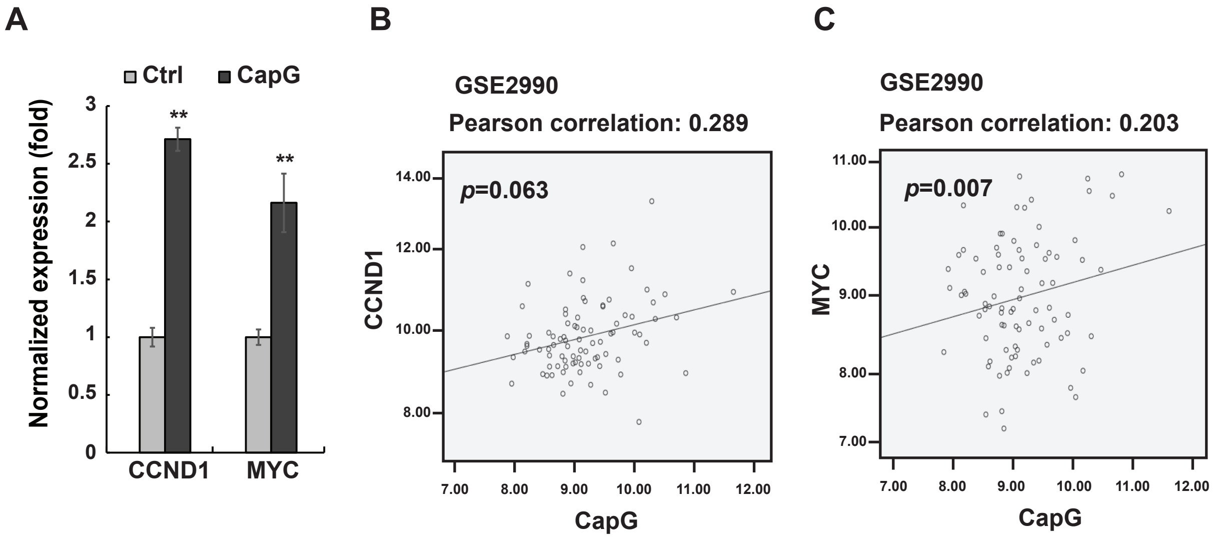
A
Ctrl
CapG
Normalized expression (fold)
3
2.5
2
1.5
1
0.5
0
**
**
CCND1
MYC
B
GSE2990
Pearson correlation: 0.289
p=0.063
CCND1
14.00
12.00
10.00
8.00
7.00
8.00
9.00
10.00
11.00
12.00
CapG
C
GSE2990
Pearson correlation: 0.203
p=0.007
MYC
11.00
10.00
9.00
8.00
7.00
7.00
8.00
9.00
10.00
11.00
12.00
CapG

sFigure 4

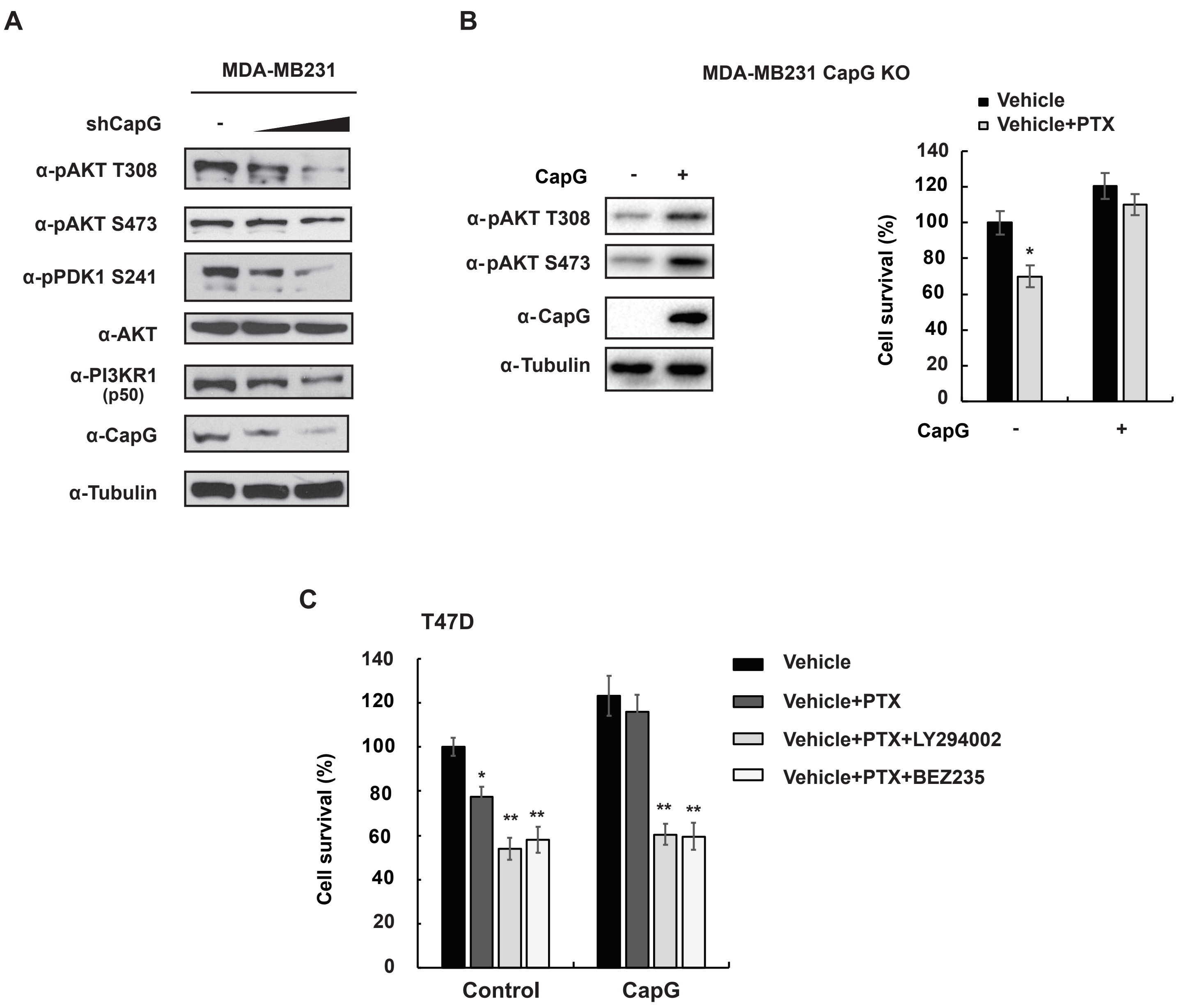

A
MDA-MB231
shCapG
-
α-pAKT T308
α-pAKT S473
α-pPDK1 S241
α-AKT
α-PI3KR1
(p50)
α-CapG
α-Tubulin
B
MDA-MB231 CapG KO
CapG
-
+
α-pAKT T308
α-pAKT S473
α-CapG
α-Tubulin
Vehicle
Vehicle+PTX
Cell survival (%)
0
20
40
60
80
100
120
140
*
CapG
-
+
C
T47D
Vehicle
Vehicle+PTX
Vehicle+PTX+LY294002
Vehicle+PTX+BEZ235
Cell survival (%)
0
20
40
60
80
100
120
140
*
**
**
**
**
Control
CapG

sFigure 5

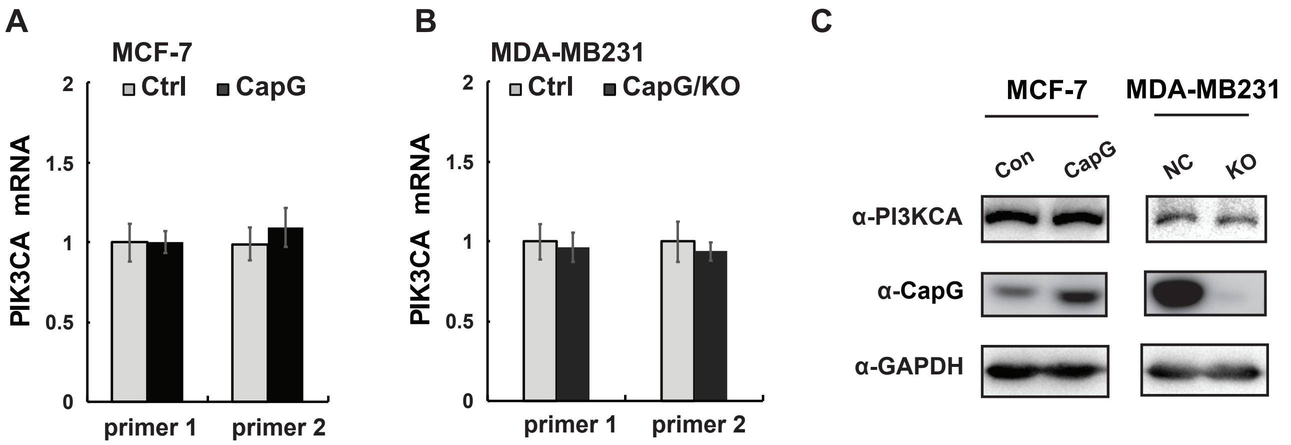

A
MCF-7
Ctrl
CapG
PIK3CA mRNA
2
1.5
1
0.5
0
primer 1
primer 2
B
MDA-MB231
Ctrl
CapG/KO
PIK3CA mRNA
2
1.5
1
0.5
0
primer 1
primer 2
C
MCF-7
MDA-MB231
Con
CapG
NC
KO
α-PI3KCA
α-CapG
α-GAPDH

sFigure 6

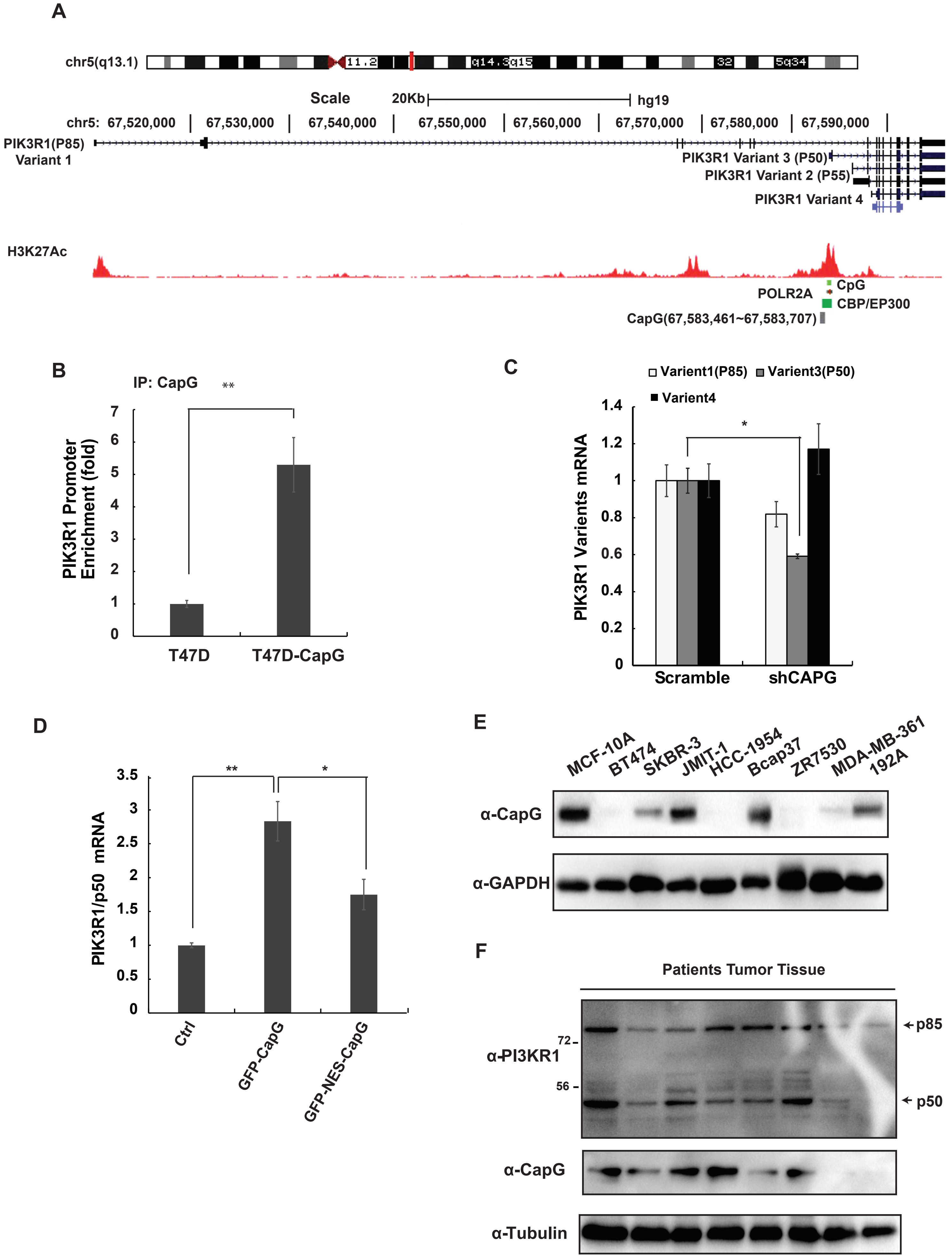

A
chr5(q13.1)
Scale
20Kb
hg19
chr5: 67,520,000 | 67,530,000 | 67,540,000 | 67,550,000 | 67,560,000 | 67,570,000 | 67,580,000 | 67,590,000
PIK3R1(P85) Variant 1
PIK3R1 Variant 3 (P50)
PIK3R1 Variant 2 (P55)
PIK3R1 Variant 4
H3K27Ac
POLR2A
CpG
CBP/EP300
CapG(67,583,461~67,583,707)
B
IP: CapG
**
PIK3R1 Promoter Enrichment (fold)
T47D
T47D-CapG
C
Varient1(P85)
Varient3(P50)
Varient4
*
PIK3R1 Varients mRNA
Scramble
shCAPG
D
**
*
PIK3R1/p50 mRNA
Ctrl
GFP-CapG
GFP-NES-CapG
E
MCF-10A
BT474
SKBR-3
JMIT-1
HCC-1954
Bcap37
ZR7530
MDA-MB-361
192A
α-CapG
α-GAPDH
F
Patients Tumor Tissue
α-PI3KR1
72
56
p85
p50
α-CapG
α-Tubulin

sFigure 7

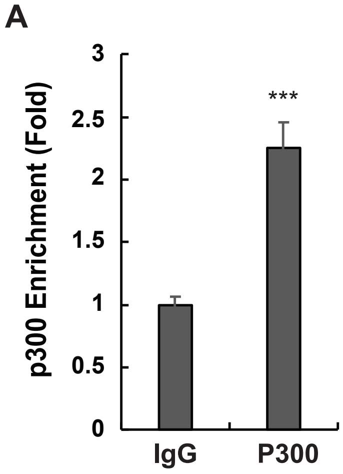

A
p300 Enrichment (Fold)
3
2.5
2
1.5
1
0.5
0
***
IgG
P300

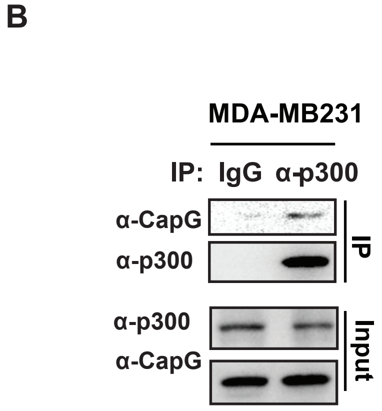

B
MDA-MB231
IP: IgG α-p300
α-CapG
α-p300
IP
α-p300
α-CapG
Input

Supplementary Table

Table S1. Relationship between CapG expression and clinicopathological features in 200 primary breast cancer patients with chemotherapy for IHC detection.

Characteristics	CapG				
	**Low**	**High**	**n**	**X^2**	***P-value***
Age（years）				0.000	1.000
<50	19	66	85		
≥50	25	87	112		
Menopausal status				0.123	0.863
Pre	18	68	86		
Post	26	87	113		
pT stage				0.710	0.701
T1	22	73	95		
T2	20	73	93		
T3	1	8	9		
pN stage				1.758	0.624
N0	28	94	122		
N1	11	30	41		
N2	3	20	23		
N3	2	9	111		
HR status				5.540	**0.026**
Negative	15	84	99		
Positive	29	71	100		
HER-2 status				3.964	**0.050**
Negative	38	111	149		
Positive	6	44	50		
Differentiation				1.410	0.296
Ⅰ Ⅱ	34	96	130		
Ⅲ	7	41	41		

HR hormone receptor; HER-2 human epidermal growth factor receptor 2；

p is based on Fisher’s exact test

Table S2. Univariate and multivariate Cox regression analyses of DFS and OS in BC patients.

Univariate and multivariate Cox regression analyses of DFS and OS in BC patients.					
**Covariates**	**Univariate a;nalysis**		**Multivariate analysis**		
	**HR (95% CI)**	***P*-value**	**HR (95% CI)**	***P*-value**	***Corrected P*-value**
DFS					
**Age (<50 versus ≥50 years)**	0.908（0.49-1.683）	0.76	——		
**T stage (T2/3 versus T1)**	1.982（1.194-3.292）	**0.008**	1.888(1.151-3.094)	**0.005**	**0.007**
**pN stage**		**0.033**	1.353(0.999-1.834)	0.051	
**N1 versus N0**	1.331(0.610-2.908)	0.473			
**N2 versus N0**	1.847(0.745-4.580)	0.185			
**N3 versus N0**	3.782(1.521-9.403)	**0.004**			
**HR (positive versus negative)**	0.873(0.479-1.589)	0.656			
**HER2 (positive versus negative)**	1.022(0.515-2.029)	0.949			
**Grade (G3 versus G1+G2)**	1.671(0.889-3.141)	0.111			
**CapG expression level (high versus low)**	3.746(1.157-12.134)	**0.028**	3.348（1.029-10.894）	**0.045**	**0.045**
OS					
**Age (<50 versus ≥50 years)**	0.453 (0.18-1.142)	0.093			
**T stage(T2/3 versus T1)**	3.804(1.930-7.499)	**<0.001**	2.728(1.475-5.043)	**0.001**	**0.002**
**pN stage**		**<0.001**	2.087(1.417-3.047)	**<0.001**	**<0.001**
**N1 versus N0**	3.219(1.129-9.182)	**0.029**			
**N2 versus N0**	4.003(1.171-13.684)	**0.027**			
**N3 versus N0**	11.940(3.757-37.945)	**<0.001**			
**HR(positive versus negative)**	0.638(0.284-1.439)	0.279			
**HER2(positive versus negative)**	1.219(0.506-2.941)	0.659			
**Grade(G3 versus G1+G2)**	2.109(0.940-4.732)	0.070			
**CapG expression level (high versus low)**	3.039(0.712-12.959)	0.133			

HR hormone receptor; HER-2 human epidermal growth factor receptor 2；

CI, confidence interval; HR, hazard ratio; DFS,disease-free survival;OS, overall survival; BC, breasr cancer.

The Benjamini-Hochberg false discovery rate analysis was used to correct for multiple comparisons.

Table S3. The clinicopathological information of 42 patients who received neoadjuvant chemotherapy with PTX regimen (18 pCR patients and 24 non-pCR patients).

Sample group	NAC regiment	NAC response	Maximum diameter of tumor(cm)	Maximum diameter of positive Lymph nodes (cm)	core needle biopsy	Phenotype
pCR-01	PCb	pCR	4.0	2.5	IDC	Her2
pCR-02	PCb	pCR	3.9	4.1	Invasive carcinoma	Her2
pCR-03	TAX	pCR	2.5	3.1	IDC	Her2
pCR-04	TAX	pCR	3.9	2.0	IDC	TNBC
pCR-05	PCb	pCR	6.5	1.0	IDC	Luminal
pCR-06	PCH	pCR	2.7	4.6	Invasive carcinoma	Her2
pCR-07	PCH	pCR	4.7	0.0	IDC	Her2
pCR-08	PCb	pCR	3.0	2.4	IDC	Luminal
pCR-09	PCb	pCR	9.0	3.3	Invasive carcinoma	Her2
pCR-10	PCb	pCR	4.0	1.5	IDC	Luminal
pCR-11	PCb	pCR	4.2	0.0	IDC	TNBC
pCR-12	PCH	pCR	3.2	2.2	Invasive carcinoma	Her2
pCR-13	PCb	pCR	6.1	3.8	IDC	Luminal
pCR-14	PCb	pCR	5.4	1.7	Invasive carcinoma	Luminal
pCR-15	PCb	pCR	3.3	4.3	Invasive carcinoma	Luminal
pCR-16	PCb	pCR	2.0	3.7	Invasive carcinoma	Luminal
pCR-17	PCH	pCR	3.7	2.4	Invasive carcinoma	Luminal
pCR-18	PCb	pCR	4.9	1.1	DCIS	Luminal
npCR-1	PCb	non-pCR	7.0	2.3	IDC	Her2
npCR-2	TAX	non-pCR	2.0	1.2	IDC	Her2
npCR-3	TAX	non-pCR	4.1	2.5	IDC	Luminal
npCR-4	TAX	non-pCR	3.4	1.6	IDC	Luminal
npCR-5	TAX	non-pCR	3.1	0.0	IDC	Luminal
npCR-6	TAX	non-pCR	4.0	2.2	Invasive carcinoma	Luminal
npCR-7	TAX	non-pCR	4.0	2.6	IDC	Luminal
npCR-8	TAX	non-pCR	5.0	3.6	IDC	Her2
npCR-9	TAX	non-pCR	2.8	1.3	IDC	Her2
npCR-10	NE-PC	non-pCR	2.4	0.0	IDC	Her2
npCR-11	PCb	non-pCR	3.1	4.3	IDC	Luminal
npCR-12	PCb	non-pCR	2.9	3.0	IDC	Luminal
npCR-13	PCb	non-pCR	3.4	3.4	Invasive carcinoma	Her2
npCR-14	PCb	non-pCR	3.2	0.0	IDC	Luminal
npCR-15	PCb	non-pCR	4.0	2.7	Invasive carcinoma	Her2
npCR-16	PCb	non-pCR	3.5	2.0	IDC	Luminal
npCR-17	PCb	non-pCR	4.5	3.0	Invasive carcinoma	Luminal
npCR-18	PCb	non-pCR	4.5	0.0	Invasive carcinoma	Luminal
npCR-19	PCb	non-pCR	3.5	3.2	Invasive carcinoma	Luminal
npCR-20	PCb	non-pCR	5.7	4.1	Invasive carcinoma	Her2
npCR-21	PCb	non-pCR	7.7	3.1	invasive micropapillar	Luminal
npCR-22	PCb	non-pCR	4.2	3.6	Invasive carcinoma	Her2
npCR-23	PCb	non-pCR	6.7	3.0	DCIS+IDC	Luminal
npCR-24	PCH	non-pCR	4.4	3.0	IDC	Her2
